# Social acceptance and perceived ecosystem services of urban agriculture in Southern Europe: The case of Bologna, Italy

**DOI:** 10.1371/journal.pone.0200993

**Published:** 2018-09-12

**Authors:** Esther Sanyé-Mengual, Kathrin Specht, Thomas Krikser, Caterina Vanni, Giuseppina Pennisi, Francesco Orsini, Giorgio Prosdocimi Gianquinto

**Affiliations:** 1 Research Centre in Urban Environment for Agriculture and Biodiversity (ResCUE-AB), Department of Agricultural and Food Sciences (Distal), Alma Mater Studiorium-University of Bologna, Bologna, Italy; 2 Department of Agricultural Economics, Humboldt-Universität zu Berlin, Unter den Linden, Berlin, Germany; 3 ILS - Research Institute for Regional and Urban Development, Dortmund, Germany; 4 Department of Agricultural and Food Marketing, University of Kassel, Witzenhausen, Germany; University of Vermont, UNITED STATES

## Abstract

Urban agriculture has become a common form of urban land use in European cities linked to multiple environmental, social and economic benefits, as well as to diversified forms (from self-production allotments to high-tech companies). Social acceptance will determine the development of urban agriculture and specific knowledge on citizens’ perception is required in order to set the basis for policy-making and planning. The ecosystem services provided by urban agriculture can be determinant in this process. The goal of this paper is to evaluate the social acceptance and the perceived ecosystem services of urban agriculture in the city of Bologna (Italy), as an example of a Southern European city. In particular, we evaluated the preferences for urban land uses, for different typologies of urban agriculture and for the resulting products, the perceived provision of ecosystem services and the willingness to engage in new initiatives. A survey that investigated these topics (including open questions, closed questions and Likert-scale evaluation) was performed on the citizens of Bologna (n = 380) between October and November 2016. Results showed that urban agriculture is widely accepted by the inhabitants of Bologna, particularly regarding vegetable production. Although intensive farming systems were the least preferred forms to be implemented in Bologna, citizens highly accepted a large variety of urban agriculture goods, with preference for those obtained from plants as compared to animal products. The willingness-to-pay for urban food products was mostly the same as for conventional ones, although the participants recognised the social values, proximity and quality of the former. Socio-cultural ecosystem services were perceived as more valuable than environmental ones. Policy-making recommendations can be extracted from the results to facilitate the development of urban agriculture plans and policies.

## Introduction

### General introduction

The significance of Urban Agriculture (UA) is grounded on its potential to address two of the major global challenges, those of responding to increasing urbanization and ensuring food security [1]. This is acknowledged by the great majority of research reports and public policies that have been issued in recent years [2]. Today, UA is being promoted for its contribution to sustainable, resilient urban development and the creation and maintenance of multifunctional urban landscapes [3]. In terms of the services provided by UA, new business opportunities are emerging [4,5] which address not only food production [6,7] but also the reduction of the environmental footprint of cities [8–10], the mitigation of and adaptation to climate change [11,12] as well as the enhancement of social and health benefits of urban and peri-urban regions [13]. Concurrently, a general rise in public concerns about the safety of urban grown foods or the absence of dedicated legislation has also been observed [14,15]. In the globally emerging research field of UA, a number of interdisciplinary European projects have addressed social issues (e.g. COST Action Urban Allotment Gardens in European Cities [16]), business opportunities (e.g. COST Action Urban Agriculture Europe [17]), lifelong learning (e.g. HORTIS–Horticulture in Towns for Inclusion and Socialisation [18]) and higher education (e.g. Urban Green Train, Urban Green Education for Enterprising Agricultural Innovation [19]).

### The evolution of urban agriculture in Europe

In Europe, UA was rooted in the traditional allotment gardens [20], which were established to cope with the living conditions (i.e., poverty, social alienation and malnutrition) arising from the population migration flow to urban areas during the industrial revolution (19th century) [20]. Under these circumstances, the so-called *migrant gardens* (or, also, *poors’ gardens*) emerged as a way to counterbalance the negative side-effects of industrialization and urbanization. Allotment gardens sprouted in lands belonging to the public administration, factories or religious communities. The availability of urban grown food from these gardens was even more important in the first half of the 20^th^ century, especially during the world wars, when towns were isolated from the countryside and food scarcity occurred [21].

The evolution of economic and socio-cultural conditions after World War II shifted the function of the allotment gardens from the original food production goal to diverse ecological-environmental, recreational, educational, social and therapeutic functions [8]. Community-owned gardens also flourished as plots of land collectively maintained by a group of people [22]. These usually originated in public green spaces where people were able to create social relationships, produce vegetables, spend their leisure time and learn. Although community-owned gardens first originated outside Europe (e.g., United States, Canada, Australia and New Zealand), from the early years of 2000 they began to emerge in Northern European countries, also as a consequence of the experienced food insecurity in urban areas associated with the economic crisis [23,24].

### Social acceptance of urban food products

Investigating the social acceptance of UA and its products is a key objective of this paper. With the emergence or recurrence of food production in cities, urban inhabitants are confronted with a new or partly unknown form of urban land use. Thus, it is not clear at the outset whether UA and its products will be appreciated by urban consumers or whether the public will be skeptical. The implementation of UA is therefore challenged by the risk of potential rejection and refusal by target groups or the general public, which is a common mechanism for any kind of innovation in its early stage of implementation [25,26].

Although the question of acceptance is highly significant–particularly for potential commercially-oriented UA businesses or social entrepreneurs–, this issue has only been addressed as a side-line by previous research on UA [27]. Only few studies, conducted in the cities of Berlin (Germany) and Barcelona (Spain), have so far specifically addressed the perception and acceptance of potential consumers in UA [3,28] or the perceptions of potential benefits, problems and risks related to UA, relying on the perspective of local key stakeholders [29–32]. Recently, Miličić et al. (2017) [33] explored the consumers’ acceptance of aquaponics products, a production system that is often recommended for application in UA. Existing studies have shown that stakeholders and potential consumers attach many benefits but also a range of risks to UA and its products. With a view to attaining a successful diffusion and communication of UA, it is therefore necessary to delve deeper into the underlying preferences and motives, which make people either accept or reject urban grown food.

### Ecosystem services of urban agriculture

The ecosystem services (ES) provided by UA have been widely theorized in the literature [34–37]. Notwithstanding that some studies have quantified certain services, such as the provision of food [38,39],a global evaluation of ES has been scarcely approached in the literature. Camps-Calvet et al. [40] assessed the ES of urban gardens in the city of Barcelona in a two-step process: identification of ES through semi-structured interviews and evaluation of the contribution of urban gardens to ES via survey. Socio-cultural services associated with urban gardens (e.g., education) were rated higher than environmental services, supporting the significance of UA in urban resilience and contestation during crises [41]. Berges et al. [7] linked the characteristics and practices of community gardens in Germany with the provision of ES. Langemeyer et al. [42] compiled available studies supporting the provision of ES by urban gardens, highlighting the importance of evaluating such benefits in urban planning and policy-making. Since ES can vary between diverse urban contexts and UA typologies, ES should be evaluated locally. Furthermore, ES have been commonly evaluated from the perspective of UA practitioners rather than of global society. This research contributes to close these gaps.

### Goal and objectives

The goal of this paper is to evaluate the social acceptance of UA in Southern Europe, where this type of urban land use is expanding. Specifically, the paper aims at:

- identifying the preferences for urban land uses, UA typologies as well as UA products;
- investigating the perceived ES by the citizens of Bologna;
- assessing the willingness to engage in new UA initiatives;
- comparing and discussing the results in a broader European context.

To do so, the city of Bologna was chosen as a case study due to its importance in Southern Europe regarding UA development and the presence of diverse on-going UA projects. Furthermore, the results are compared to the outcomes of a similar study conducted in Northern Europe (Berlin, Germany) [3].

## Materials and methods

### Case study: Bologna

**Fig 1:**
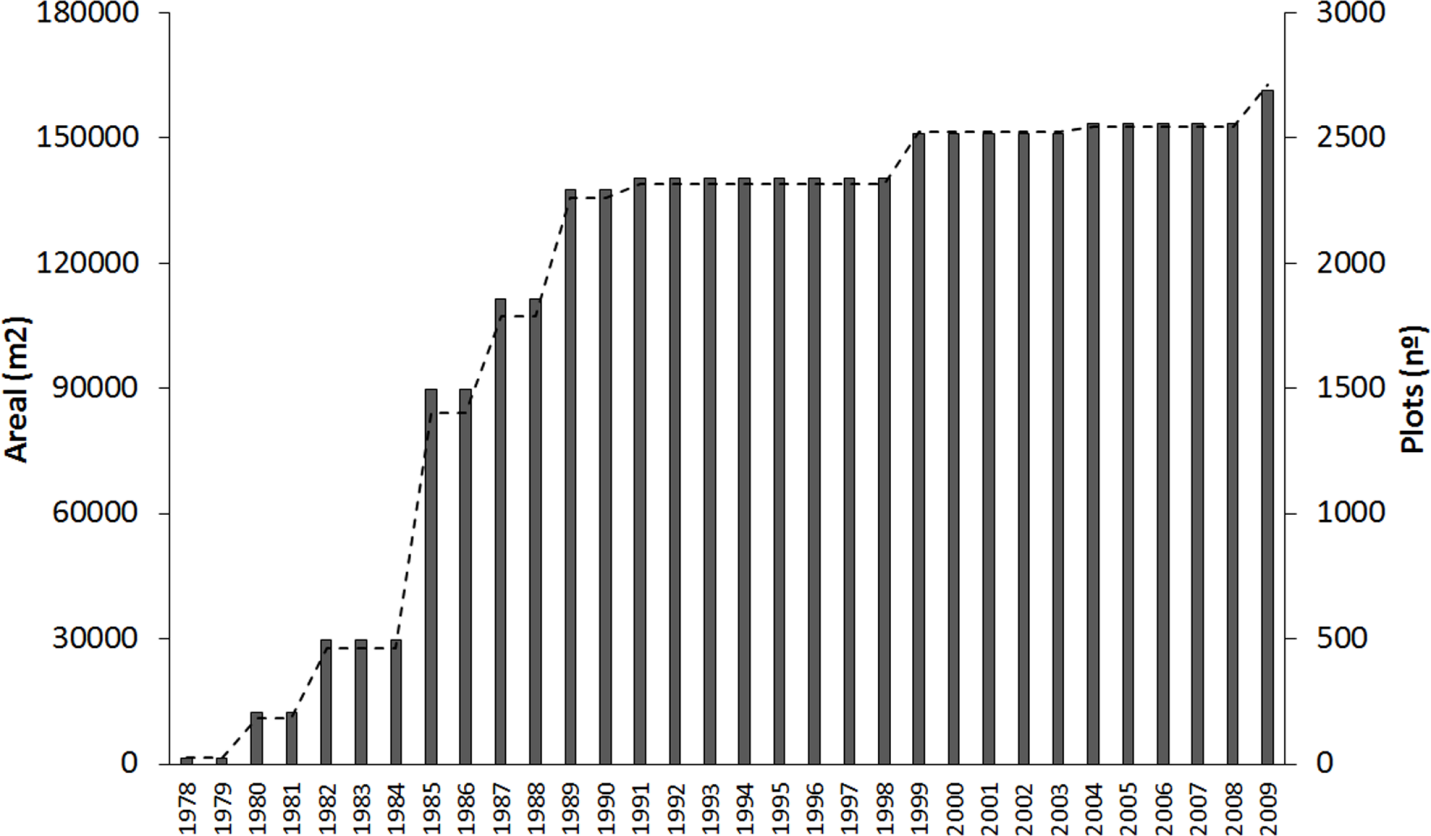
Timeline: Evolution of UA in Bologna (data including official public vegetable gardens only).

Bologna is the capital of the Emilia-Romagna region and the seventh most populated city in Italy, with a metropolitan population of approximately 400,000 inhabitants over a geographical area of 140 km^2^ [43]. Eight percent of the urban territory is devoted to green areas, resulting in a green area of about 30 m^2^·per capita, overall reflecting the national average values. Bologna has been a frontrunner in Italy regarding UA, with many initiatives developed in the last 40 years that resulted in a rapid growth of both the area devoted to UA and the number of allotments (Fig 1).

Urban gardens in Bologna encompass not only municipality-supported (e.g., allotments) but also grassroots experiences (e.g., food co-ops) [44]. While the number of municipal allotment gardens stagnated in recent years (2,700 gardens are currently accessible to city inhabitants of all ages), the diversity of UA initiatives has sprouted all over the city, including guerrilla gardening on abandoned flowerbeds as well as gardens as a requalification activity on abandoned industrial neighborhoods [44]. Furthermore, the city council supported the creation of the first rooftop community garden in a social housing building in Italy [38]. According to a recent survey [45], urban agriculture in Bologna extends over around 29 ha, including urban gardens (16 ha) and other garden typologies (e.g., private gardens, gardens in monasteries, schools, illegal squatted gardens) (13 ha). The same survey indicated that urban gardeners in Bologna are mainly males (69%), Italian citizens (94%), and generally above 60 years of age (77%). In addition to those citizens already involved in UA, the waiting list for municipal gardens includes more than 4,600 requests, of which 43% are from young urbanites (<40 years old), while the rate of women (47%) and foreign citizens (10%) have both increased [45].

UA in Bologna has recently become a political issue, resulting in the creation of the international design competition *Orti per Tutti (Gardens for all*), where architects, landscape designers and horticulturists were called to provide a key design for the future gardens of the city [46]. In 2015, Bologna also signed the Milan Urban Food Policy Pact, which enhances the implementation of food policies towards sustainability and social justice [47]. In 2017, the International Symposium of Greener Cities for More Efficient Ecosystem Services in a Climate Changing World took place in Bologna [48], and the International Horticultural Exposition, which will focus on City Regeneration and Urban Agriculture, will be hosted in 2019 [49].

### Data collection

To explore the UA preferences of the inhabitants of Bologna, the present study applied a quantitative approach by implementing a survey on a probabilistic sample. The target population were citizens of Bologna who were adults (≥18 years) and had lived there for at least two years. Since Bologna has six well-defined neighborhoods and 18 sub-neighborhoods, a two-stage cluster sampling for recruiting was used [50,51]: (a) the data collection areas were randomly chosen and (b) the participants were randomly invited to join the study.

First, a random generator was used to identify the sub-neighborhoods where data collection should be performed. The proportional ratio of inhabitants was introduced in the random generator and 10 sub-neighborhoods out of 18 were randomly chosen. One sub-neighborhood was chosen two times and replaced by the 11th option. This approach assured the same chance to participate in the survey for every inhabitant of Bologna. For each of these sub-neighborhoods, highly-dense public spaces (e.g., municipal markets, squares, public services) were identified as recruitment spots.

As a second randomization step, the interviewers addressed every third passerby in the recruitment spots to minimize the potential selection bias by the interviewer. The data collection was conducted at hotspots of the chosen neighborhoods between October and November 2016 (e.g., main squares). The participants were recruited via a “paper and pencil questionnaire” that was handed out to the interviewee by the interviewers. The questionnaire used for this research study (S1 File) followed the same structure as the one employed in a previous survey for the city of Berlin [3,28]. It is composed of 7 topical sections and contained item batteries about the preferences, perception and attitudes of the inhabitants towards the usage of green and open spaces in the city, the production of agricultural goods, the resulting products from UA and about the socio-cultural and environmental ES. The survey closed with questions about the socio-demographic characteristics of the participants.

**Fig 2:**
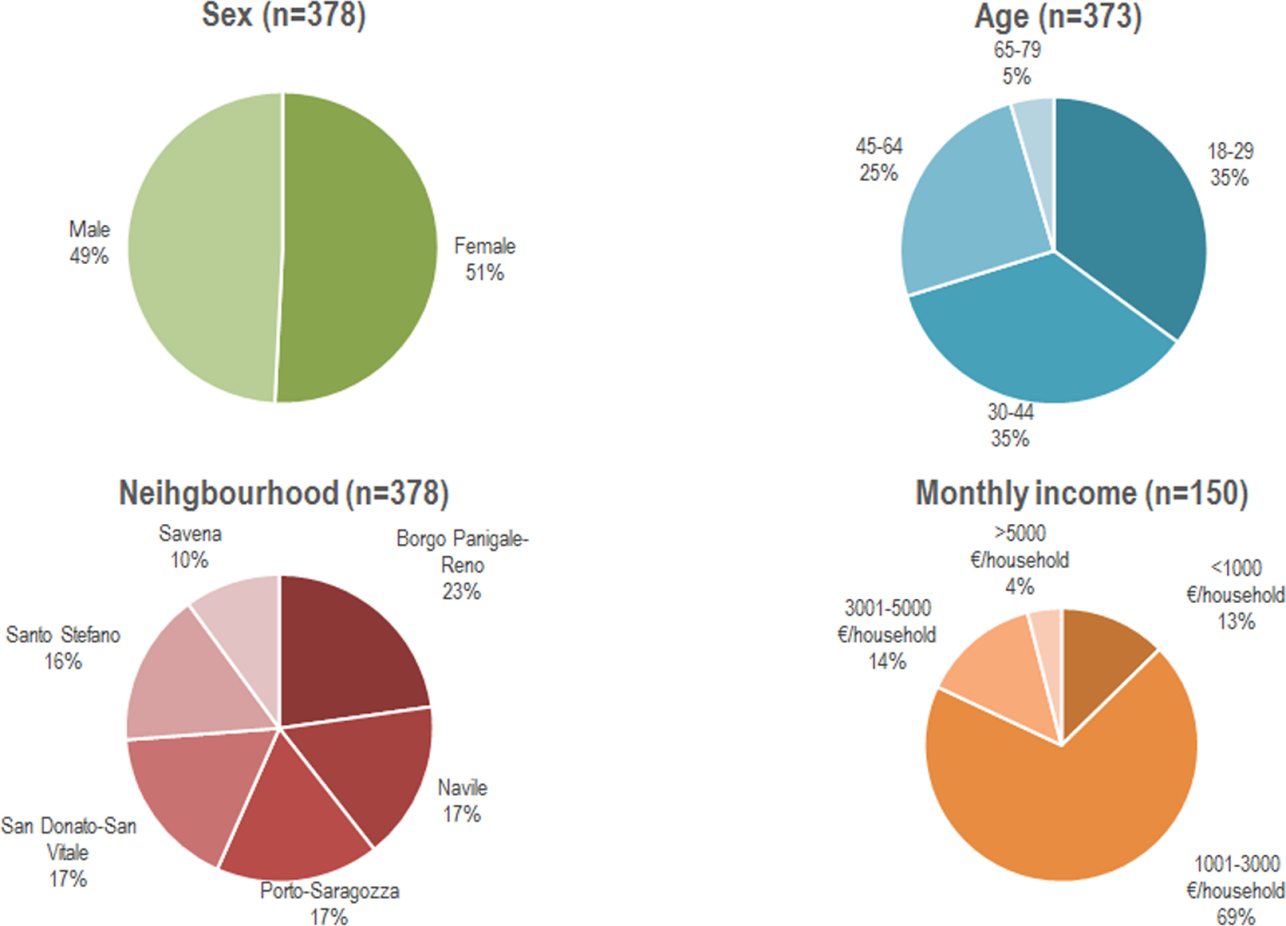
Characteristics of the sample in terms of sex, age, neighborhood and monthly income.

In total, the sample consisted of 380 completed interviews—one participant decided to drop out of the interview -, leading to a confidence level of 95% and a confidence interval of five for a population of 390.000 inhabitants [51]. Fig 2 shows the characteristics of the sample.

Even though a probability sampling was used in this study, the resulting sampling was not representative of the Bologna population for all the attributes, according to socio-demographic statistics [52]. The sample was representative for sex structure (Sample: Male 49%, Female 51%; Bologna: Male 47.3, Female 52.7) and no significant difference (*p* = .245; binomial test) was found when compared to the Bologna population. Although the similarity of monthly income attribute between the sample and the actual population cannot be calculated as income categories were used in the questionnaire, most of the participants indicated that their average monthly income was in the range of 1001–3000 Euros per month, which is representative of the average income of the Bologna population (1692.25 Euros per month). On the other hand, the age structure of the sample was not representative of the Bologna population (One-sample Chi^2^-Test: p < 001). The younger age groups were over-represented in the sample (18–29: Sample 35%, Bologna 14.8%; 30–44: Sample 35%, Bologna 25.08%), to the detriment of the older age groups, which were under-represented (45–64: Sample: 25%, Bologna 31.2%; >65: Sample: 5%, Bologna 29.1%). However, this difference between the official statistics and the sample can be explained by the temporal population of Bologna, as a university town, with a high rate of incoming students and recent graduates in their first jobs. Collected data is displayed in S2 File and S3 File.

### Evaluation of ecosystem services

As part of the questionnaire, the ES of UA were assessed in terms of the impact on the environment and society (Annex 1, See questions 10 and 11). The participants were asked to rate their agreement level regarding the contribution of UA to specific environmental and socio-cultural ES. The list of evaluated ES was based on the classification of ES introduced by The Economics of Ecosystems & Biodiversity [53] and the classifications used in ES studies on urban gardens, home gardens and urban parks [7,40,54–57]. The list of ES considered 14 environmental ES and 12 socio-cultural ES.

### Data analysis

Data analysis depended on the type of question. The closed questions (e.g., knowledge on UA, preferences of usage of green space, market options, ES and citizen engagement) were first evaluated through descriptive statistics. Afterwards, bivariate statistics were employed to observe the association of these results with sociodemographic attributes (i.e., age, gender and income), including either chi”-tests, Mann-Whitney-U-Tests or Spearman correlation. The test statistics U (Mann-Whitney-U-Test), x^2^ (Chi”-Test) and r_s_ (Spearmen correlation) are reported in the text when correlation was significant (p < .05), the effect size “r” was calculated for the Mann-Whitney-U-Test to evaluate the impact of these attributes. Non-parametric tests were chosen due to the lack of normal distribution in the data and the ordinal scale of the variables. Due to the large amount of statistical results, only significant ones are shown in the text. As an open question, the definitions of UA provided by the respondents were manually coded to apply a network analysis with the aim of exploring patterns and observing relations between the employed concepts using the software Gephi 0.9.2 [58].

### Ethics statement

The potential respondents were informed about the scope of the study and a consent question for those willing to participate was included at the beginning of the questionnaire. To ensure anonymity of the participants, personal data such as name, e-mail address or physical address were not collected. The procedure was revised and approved by the Research area and the Ethics committee of the University of Bologna, following the Ethics guidelines of the H2020 program of the European Commission.

## Results -The social acceptance and perceived ecosystem services of urban agriculture in Bologna

This section describes the results of the survey for each section: knowledge, preferences for UA typologies, acceptance of UA products, evaluation of ES and participation. In general, results showed a high acceptance of UA initiatives and products in Bologna, suggesting that new UA projects, including business-oriented ones, would succeed in their establishment.

### Knowledge and concepts of urban agriculture

**Fig 3:**
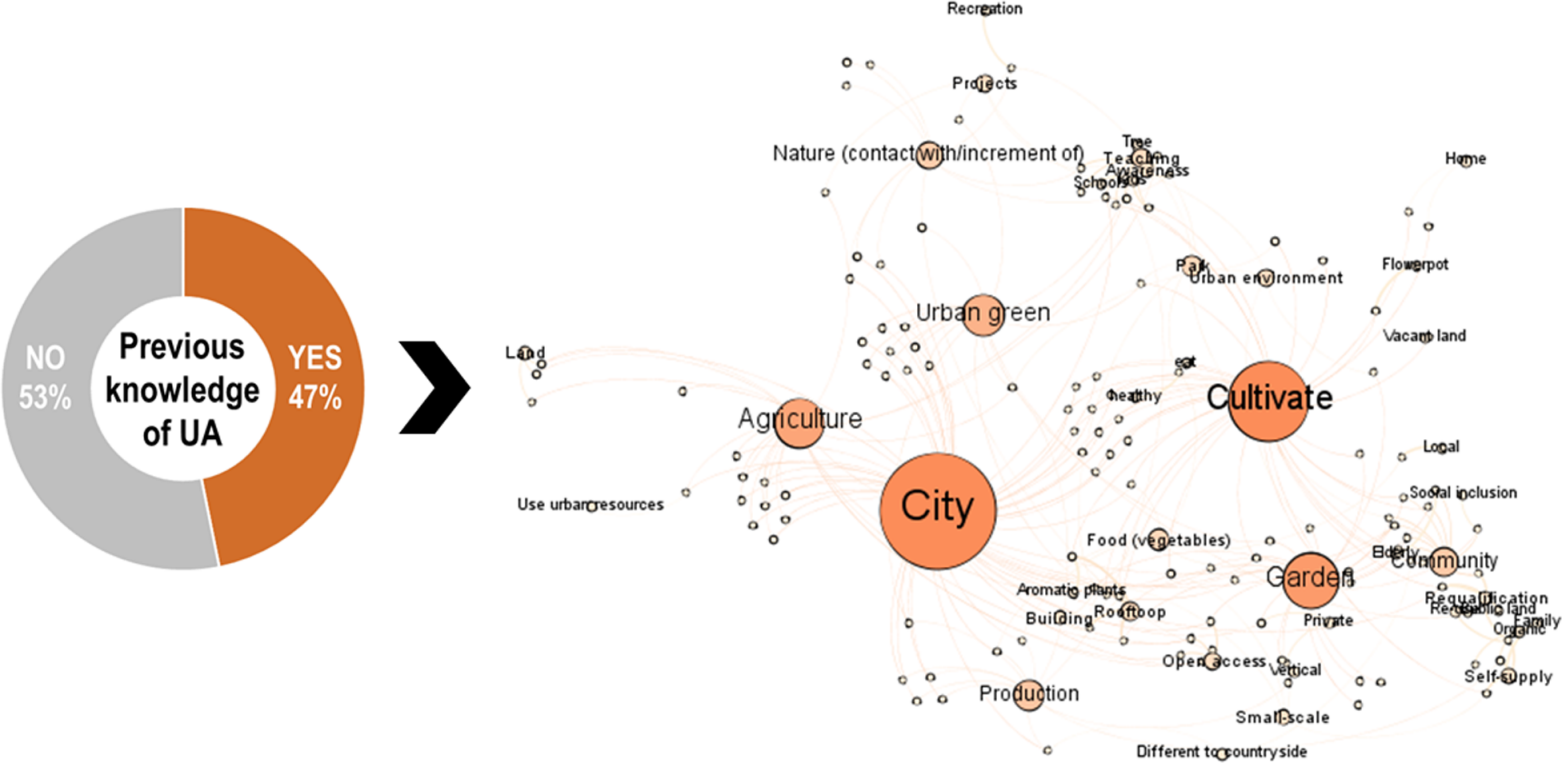
Participants’ previous knowledge on UA [Q2]: *Have you already heard about the term „urban agriculture*?*”* and network of the concepts employed for the definition of UA [Q3] “*How would you explain/define UA*? *(for participants with previous knowledge)* and [Q3b]: *What do you think UA is like*? *(for participants without previous knowledge)*.

The results revealed that the concept of UA is still rather unknown among the residents of Bologna as more than half of the surveyed participants expressed that they had not previously heard about UA (Fig 3). Irrespective of whether the participants had previous knowledge of UA, they were requested to express what they imagined UA to be. The network of concepts used in their definitions (Fig 3) shows that the citizens of Bologna highlighted the urban element of UA, the concept of *city* being the most employed by the respondents (46.3%). The *cultivation* (30.9%), *production* (9.6%) or creation of a *garden* (20.3%) in an urban context were understood as the integration of *agriculture* (17.9%) into cities. Three main benefits were strongly linked to UA: the increase of *urban green spaces* (14.6%) and *parks* (5.7%), a higher *contact with nature* (8.1%) and the *educational* opportunity of UA (4.9%), particularly for *kids* and *schools*, which contributes to the *environmental and food awareness* of citizens (3.3%). The respondents described a diversified and multifunctional UA, where the *community* plays an important role (8.9%) but where individual actors can also participate, in different urban spaces (e.g., *rooftop*, *buildings*, *vacant areas*), while offering multiple benefits (e.g., *self-supply*, *urban requalification*, *social inclusion*, *recreation*). About 4.1% believed that UA might be an *accessible* space of cities.

Data analysis showed no significant association between having previous knowledge on UA and the sociodemographic characteristics of the participants. Chi”-Test showed no significant correlation between previous knowledge on UA and the gender of the participant (x^2^ (1) = .036, *p* = .85). Mann-Whitney-U-Tests showed no significant correlation between previous knowledge and income (U = 2,501, *p* = .288) or age (U = 17,184.5, *p* = .928).

### The potential implementation of urban agriculture—Preferences for uses of green urban space, different UA types and production orientation

This section explores the preferences of the inhabitants of Bologna regarding different types of productive urban land uses in general, represented by the question [Q4a]: *“Which of the following uses of green and open space would you like to have in your living surrounding*?*”*. The question aimed to determine whether the participants consider UA as a valuable form of urban land use in comparison to other uses of urban green spaces, such as parks or private gardens. According to the results, public parks (1.15) and urbans gardens (between 1.3 and 1.4) were highly accepted by the respondent, who showed a strong rejection of meadows (3.3 out of 5 as maximum rejection). Among the diversity of publicly accessible gardens, residential gardens and intercultural gardens were rated higher than gardens with a demonstrative character (e.g., maize labyrinths or educational trails).

**Fig 4:**
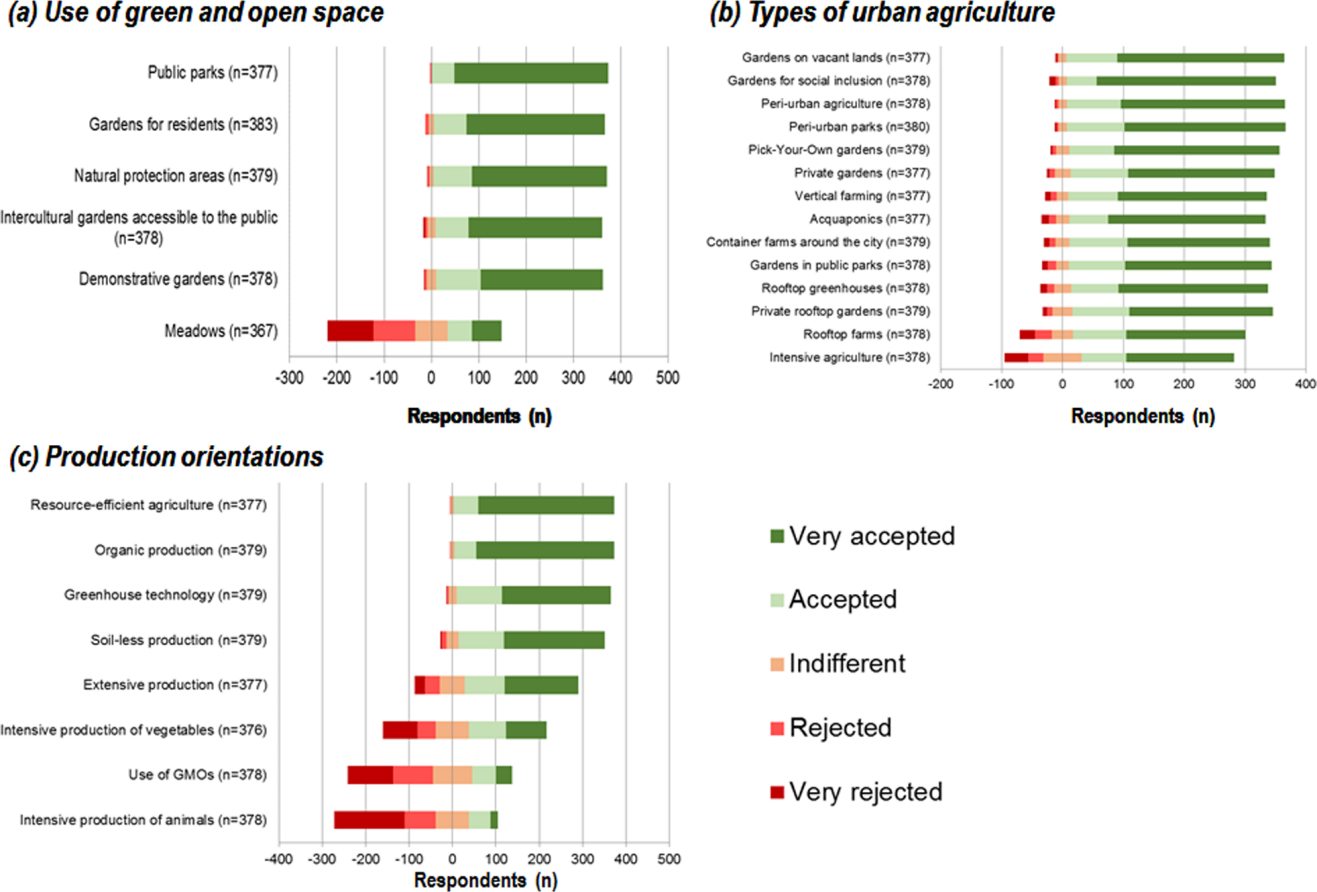
Social acceptance of uses of open and green spaces in the city of Bologna [Q4a], types of UA [Q4b] and production orientations [Q5].

Besides exploring the general preferences for urban land use, we asked participants about their acceptance and approval of different UA types. The types in question ranged from low-tech to high-tech solutions of UA, embracing different levels of market orientation, production intensity and location. The results revealed that the participants highly appreciated UA on vacant land, as well as gardens for social purposes and activities taking place in the peri-urban areas. Aquaponics and vertical farms had a higher level of acceptance than growing activities on urban rooftops (such as rooftop gardens or rooftop greenhouses). Nevertheless, all the proposed UA types were broadly accepted and over 50% of the participants highly appreciated them, with intensive agriculture reaching the lowest level of acceptance (Fig 4B).

To explore participants’ views on different production orientations, we asked about the level of acceptance of various potential UA production orientations (e.g., organic, intensive, or genetically modified organisms (GMOs)) [Q5]. The results demonstrated that environmentally friendly production orientations, such as resource efficient or organic production were well supported, whereas intensive crop production, the use of GMOs and the intensive production of animals were strongly rejected (Fig 4C).

The statistical analysis of the sociodemographic attributes of the participants showed that the preference for peri-urban parks and pick-your-own gardens as potential UA typologies was significantly higher in the female group. However, the effect size (r) was very weak for both results according to the Mann-Whitney-U-Test (Peri-urban Parks: U = 15,999, p = .45, r = .103; Pick-your-own gardens: U = 15857.5, p = .048, r = .102). For the variables income and age, Spearman correlation test showed that the preference for public parks and extensive production was significantly related to income. People in higher income groups had a higher preference for public parks (*r*_s_ = -.242, *p* = .003) and a lower preference for extensive production (*r*_s_ = .178, *p* = .030). Regarding age, older participants preferred intercultural gardens (*r*_s_ = -.122, *p* = .019) and gardens on vacant land (*r*_s_ = -.137, *p* = .008) more than younger participants. However, a closer look at the effect sizes (r_s_) indicated that there are only small effects of the sociodemographic attributes on the preferences.

### Market options for urban agriculture products

The following section focuses on the acceptance of potential products from UA, the reasons why citizens may prioritize UA products, their willingness to pay for these products and the potential risks associated with products from UA. First, the participants were asked about their knowledge, approval and willingness to buy a diversity of UA products [Q6]. The results (Table 1) illustrate that the participants generally showed a high willingness to buy products from UA. Seedlings, specialty products and horticultural products (both fruits and vegetables) as well as aquaponics products and honey were highly accepted, with over 80% of the participants claiming they would be willing to buy them. The lowest purchase willingness levels were expressed for animal products (e.g., eggs, milk, wool and meat). In general, the respondents showed high levels of acceptance and knowledge of UA-products (>75% for all products).

**Table 1:** Participants willingness to buy products from UA, acceptance and knowledge; percentage of Yes among total respondents (n) [Q6].

UA products	Willingness to buy		Acceptance		Knowledge	
	Yes (%)	*n*	Yes (%)	*n*	Yes (%)	*n*
Seedlings	92.6	171	94.8	253	93.9	230
Specialty products	91.9	180	92.4	288	82.3	195
Fruits	91.6	175	93.6	238	90.9	241
Greenhouse vegetables	89.8	163	97.6	251	95.3	253
Aquaponics	86.8	186	90.6	303	61.9	160
Honey	86.5	188	89.7	283	79.1	173
Open-air cultivated vegetables	83.4	165	90.2	249	97.4	247
Eggs	76.5	177	82.1	249	82.1	195
Milk	73.0	176	77.9	235	92.8	219
Wool	70.1	171	81.5	300	52.5	148
Meat	69.4	177	76.0	246	78.8	197

**Fig 5:**
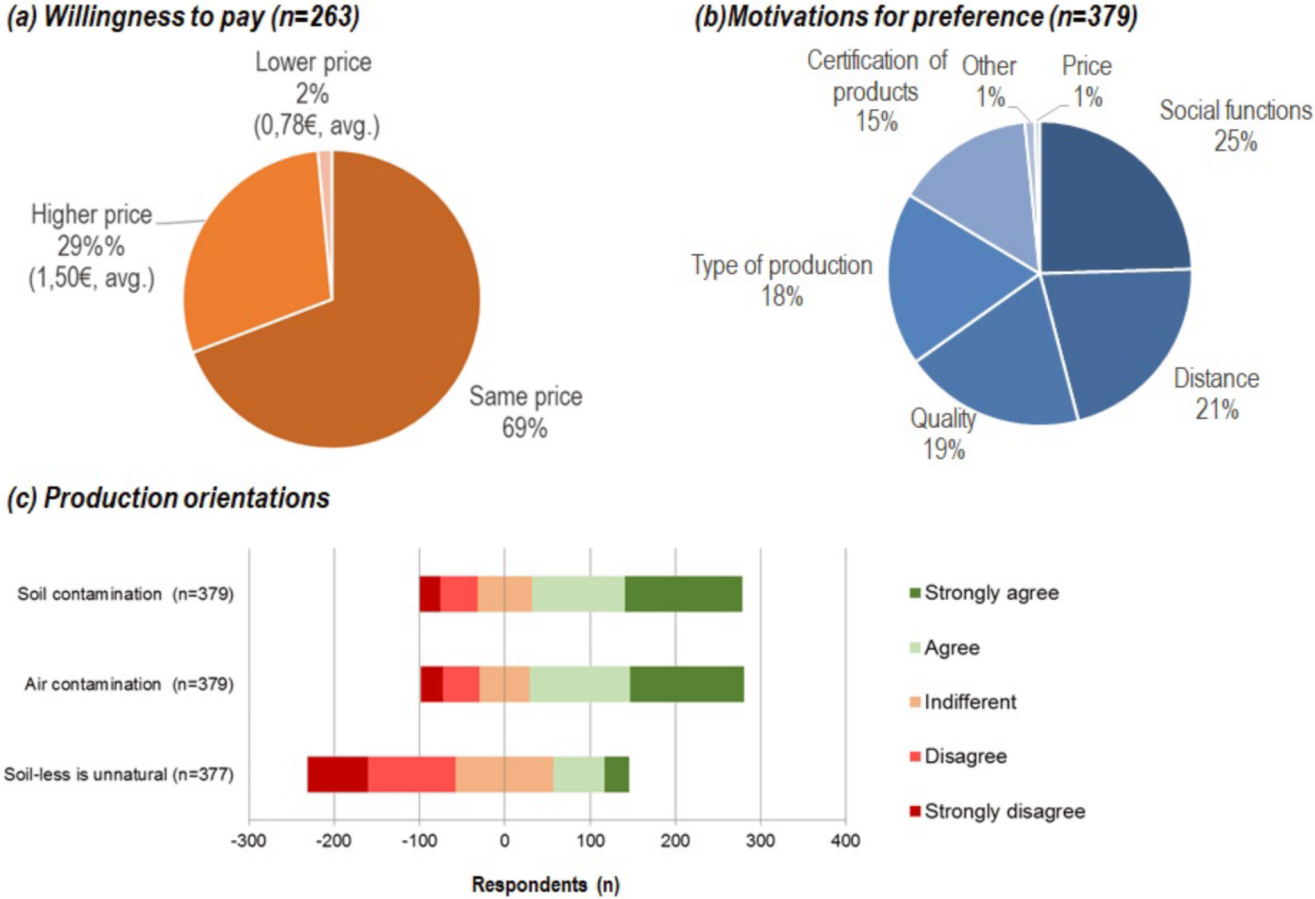
Willingness to pay for products from UA [Q8], participants’ reasons for preferring urban products to conventional ones [Q7] and perception of risks attached to the potential UA products [Q9].

Despite the high willingness to buy UA products, the willingness to pay for such products was not significantly higher than for conventional agricultural products. While only around 30% of the participants would be willing to pay more for UA products –150% price, on average–, the majority expressed that they would either pay the same price (69%) or even less (1%)– 78% price, on average–(Fig 5A). The motivations to prefer products from UA over conventional food products were diverse. While some participants appreciated UA products due to their social functions, others would prefer them because of lower transport distances or the quality of the products (Fig 5B). Finally, one major hindering factor for the acceptance of UA products were the concerns for potential risks. Regarding perceived risks, over 60% agreed to the statements related to risk of pollution (both from air and soil contamination). A minor part of the participants considered soilless cultivation practices as an ‘‘unnatural” way of producing (Fig 5C).

The data showed no significant influence of gender (U = 9,492, p = .070), age (*r*_s_ = -.076, *p* = .423) and income (*r*_s_ = .003, *p* = .956) on the willingness to pay. Concerning the risks associated with UA products, female participants were less concerned about air contamination (U = 20,835.5, p = .003, r = -.150) and soil contamination (U = 20,879.5, p = .003, r = -.153). The higher income groups significantly perceived less risk due to air contamination (*r*_s_ = -.165, *p* = .043) than the lower income groups.

### Perceived ecosystem services of urban agriculture

**Fig 6:**
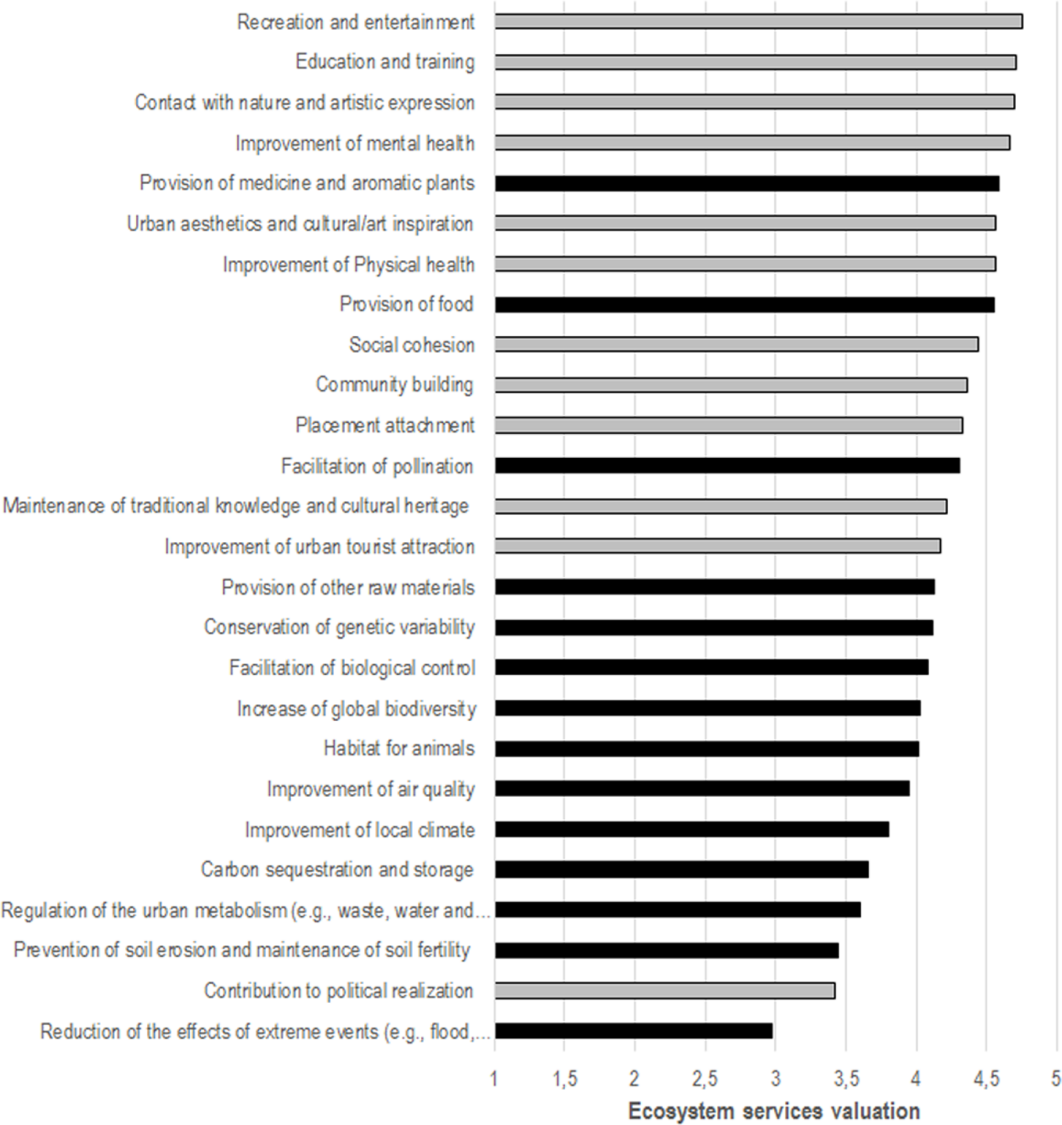
Evaluation of the ES provided by UA—from highest contribution (5) to lowest contribution (1)—for environmental services [black] [Q10] and socio-cultural services [grey] [Q11].

Respondents were asked to assess the ES of UA as environmental services [Q10] and socio-cultural services [Q11]. The participants rated the socio-cultural services higher than the environmental services (Fig 6). In particular, “Recreation and entertainment”, “Education and training” and “Contact with nature and artistic expression” were the most valued services. Regarding environmental services, the “Provision of medicinal and aromatic plants”, “Provision of food” and “Facilitation of pollination” were at the top of the list. On the contrary, the ES with the lowest values were “Reduction of effect of extreme events”, “Contribution to political realization” and “Prevention of soil erosion and maintenance of soil fertility”. In general, the respondents highlighted the contribution of UA to environmental and socio-cultural services, 73% of which obtained values above 4 (Agree). Only one ES was rated below 3 (Indifferent).

Gender differences in the valuation of ES were found for three ecosystem services. The female level of evaluation was significantly higher than that of the male participants for “Improvement of local climate” (U = 13,215.5, p = .025, *r*_s_ = -.120), “Contact with nature and spiritual experience” (U = 15,606.5, p = .010, *r*_s_ = -.132) and “Urban aesthetics and cultural/art inspiration” (U = 14,579.5, p = .018, *r*_s_ = -.126). For all three of these the effect size (*r*_s_) was small. While age had no significant influence on the evaluation of ES, income influenced the evaluation of “Education and training” (*r*_s_ = -.177, *p* = .031) and “Contribution to political realization” (*r*_s_ = -.229, *p* = .012), which was lower among the higher income groups.

### The role of urban agriculture in the city

**Fig 7:**
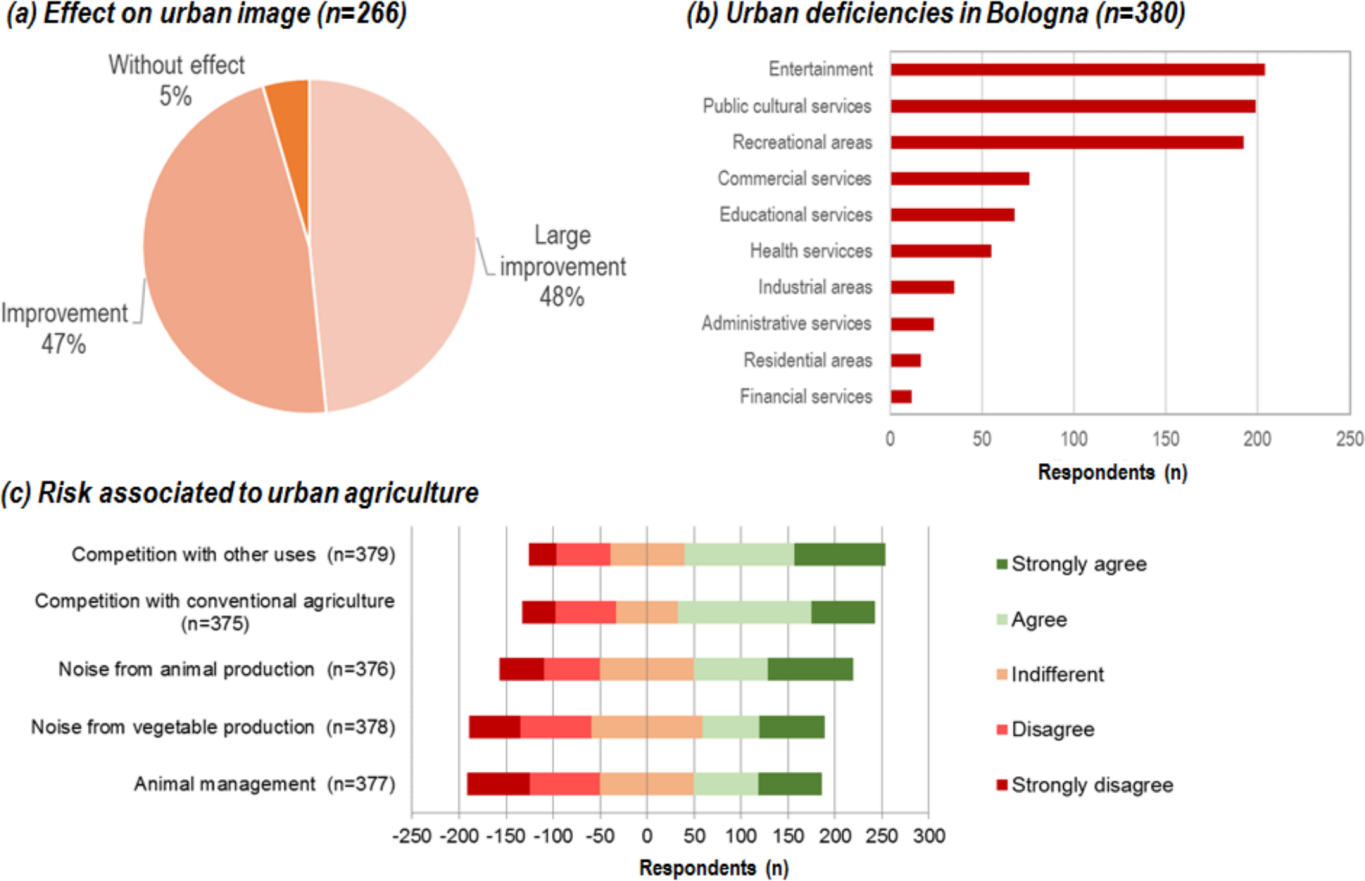
Perception of respondents regarding the effect of UA on urban image [Q12], the deficiencies in Bologna [Q1] and the risks associated with UA as a system [Q9].

The inhabitants of Bologna were asked to evaluate the potential effect of UA on the urban image of the city [Q12], identify the urban gaps in their neighborhoods [Q1] and assess potential risks of the system [Q9]. About 95% of the participants indicated that UA would improve or greatly improve the image of Bologna (n = 266) (Fig 7A). In particular, none of the respondents indicated that UA would worsen the image of the city. New UA initiatives would fill the urban gap of recreational areas identified by the respondents as the third most urgent deficiency (Fig 7B). The respondents agreed with the fact that UA has some associated risks and more than half supported the five proposed statements. The most important risk was the competition with other roof uses (e.g., photovoltaics) and with rural farmers (Fig 7C).

Concerning the risks that are associated with UA, statistical tests showed that the female participants perceived less risks of noise from vegetable production (U = 19,958.5, p = .033, r = -.110) and noise from animal production (U = 19,765, p = .032, r = -.111). All calculations indicated a low effect size. While age had no effect on the risk perception, the higher income groups saw significantly less risk from competition with other roof uses (*r*_s_ = -.195, *p* = .017), animal management (*r*_s_ = -.182, *p* = .026) and noise from vegetable production (*r*_s_ = -.165, *p* = .044) than the lower income groups.

### Engagement of citizens in urban agriculture initiatives

**Fig 8:**
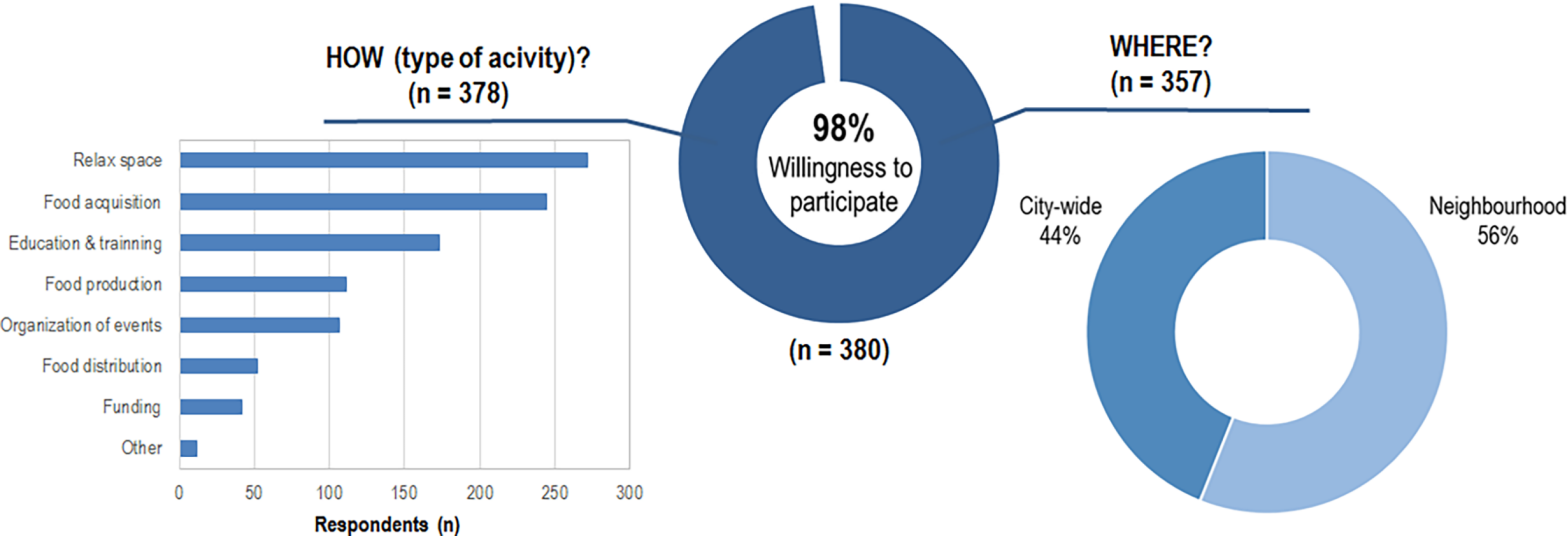
Potential engagement of interviewees in UA projects: Preferred type [Q13] and location of activities [Q14].

The citizens of Bologna showed a high engagement in UA initiatives and 98% of the respondents were willing to participate (Fig 8). Using the agricultural spaces as a recreational area and becoming customers of the food produced were the preferred participation paths. On the contrary, becoming distributors of the food products or providing funding for the initiatives were the least chosen options. Engagement in UA initiatives in the same neighborhood was slightly preferred (56%) than in other areas of the city (44%).

There was a significant association between gender and the respondents’ willingness to participate in education and training x^2^ (1) = 9.758, *p* = .002. Based on the odds ratio, the odds that women would participate in education and training on UA was 1.92 times higher than the odds that men would participate. Similar results were found for funding UA projects (x^2^ (1) = 8.050, *p* = .005), where the odds that women would fund projects were 2.69 times higher than for the male participants of the survey. However, the effect size was low for both calculations (Education and training: .159; Funding: .144). While income had no effect on the results, age was significant. The younger participants had a significantly lower willingness to participate in UA by producing food than the older age groups (U = 11,487, p = .001, r = .171). The results indicated a small effect size.

## Discussion

### The acceptance of urban agriculture in Bologna

Despite the growing importance of UA worldwide in recent years [13], more than half of the surveyed participants were not aware of it (Fig 3). This is also reflected in a greater acceptance of more traditional typologies of rural agriculture adaptation to the urban context (e.g., traditional on-soil gardens, organic farming protocols), rather than highly engineered urban horticultural solutions (e.g., rooftop farms) (Fig 4B) [59]. Although the city of Bologna hosts the pioneering experience of the social inclusive rooftop garden of via Gandusio (cited in several scientific reports, magazines and TV shows) [38,44,60], apparently the social acceptance of the potential productive function of rooftop agriculture is still limited, as also evidenced in a comparative study in Barcelona and Berlin, where respondents claimed a number of perceived risks associated with this technique (e.g., conflict with traditional agricultural imaginary, gentrification potential, scarce awareness of soilless growing techniques, competition with alternative rooftop uses and limited organic certification) [31].

Concerning the participation in UA, respondents were highly willing to commit themselves (98%, Fig 8), although mainly to initiatives within their own neighborhood, thus confirming that the geographical distribution and long-term sustainability of UA initiatives in cities is inversely related to the distance from home [61]. Overall, despite the fact that respondents showed a propensity toward purchasing UA products (Table 1), the majority (69%, Fig 5) would not pay any premium price for them, similarly to previously published results [33]. Lastly, participants stated they would economically appreciate the social function (25%) and the reduced travel distance (21%) of urban grown products [62].

The acceptance of certain urban animal products was also high (over 76% for all categories) and highest in honey and aquaponic products. Urban honey production is currently facing an exponential growth, which is mainly associated with the growing public debates about bees and beekeeping, the diversification of beekeeping opportunities, and new actors and motivations for beekeeping [63]. In a recently published web survey on the acceptance of aquaponics [33], half of the respondents were unaware of this technique for simultaneously growing fish and vegetables. However, once the main principles of aquaponics were described, half of the participants considered it a possible way for pursuing a more sustainable food production. Consistently, the perceived environmental sustainability of aquaponics was the second most cited motivation by practitioners in an international survey [64].

The sustainability of food production was also highly appreciated by the survey participants in Bologna, who mainly referred to it with the concepts of resource efficiency and organic production (Fig 4) [65,66], whereas the adoption of GMOs was not acceptable by most of the participants, mirroring more general trends [67]. On the other hand, the risk of contamination, a subject explored in recent studies in Bologna [15,68], was considered as a main drawback to the diffusion of UA, as also evidenced in previous studies [69,70].

### Comparing the acceptance of UA in Berlin and Bologna

In this section, we compare the results obtained in the city of Bologna with the previous study of Specht et al. [3] in Berlin. The inhabitants of Berlin were more aware of UA (60% had previous knowledge) than in Bologna (47%), probably due to the high diversity and number of UA projects in the German city, particularly in emblematic spaces like the former Tempelhof airport (http://www.thf-berlin.de/).

Public parks were the preferred urban green infrastructures in both cities. On the other hand, the inhabitants of Bologna rejected animal-related landscapes and meadows, whereas Berliners refused the high-tech and intensive UA options (e.g., aquaponics) the most. Although the implementation of rooftop gardens was the preferred option in Berlin, they showed a lower level of acceptance in the city of Bologna, where forms of intensive UA were the least valued options. On the contrary, the citizens from both cities agreed in the preference for resource-oriented and organic farming and in the rejection of GMOs and intensive livestock production, highlighting the prevalent environmentally-friendly discourse of UA in Europe.

Concerning the market options for UA products, Berliners showed a lower willingness-to-buy (22–50%) than the Bolognese (69–92%). Vegetables were the preferred product in Berlin, while seedlings and specialties were prioritized in Bologna. Nevertheless, some animal products (i.e., meat, wool, milk) were the least preferred options in both cities, underlining the low acceptance of animal production in UA. While the most important requirement for UA products in Berlin were the production protocol (i.e., organic) and the high quality, Bologna’s inhabitants appreciated more the social benefits and the local nature of UA. In both cities, the food safety risk due to urban pollution was considered significant.

As for the contributions of UA to the city and its citizens, the improvement of the urban image was highlighted in both studies. While Berliners most valued the potential environmental improvements resulting from UA, Bologna citizens placed in the foreground the social benefits associated with the new uses of urban spaces (e.g., education, recreation). These results suggest that while the environmentally-friendly discourse dominates in Berlin, the socially-oriented UA is also in the spotlight in Bologna.

### Evaluating the ecosystem services of urban agriculture

The evaluation of the ES of UA by the citizens of Bologna showed some similarities and some divergences when compared to a recent study of urban gardens in Barcelona (Spain) [40]. First, socio-cultural ES had a significant position in both studies. In the most ranked services, both studies highlighted the role of UA in the “Contact with nature” and “Improved mental health”. Regarding socio-cultural ES, the ranking was similar in both studies with some minor variances. At the same time, the political realization of practitioners of UA was the least valued socio-cultural ES in both studies.

However, several differences were found in the evaluation of environmental ES. While the provisioning services (food, medicinal and aromatic plants) were the least valued environmental ES in Barcelona, they were the highest ranked by the citizens of Bologna. However, while food provision was more valued than medicinal and aromatic plants provision by the practitioners of Barcelona gardens, the opposite was observed in the Bologna study. In a similar vein, prevention of soil erosion and maintenance of soil fertility was the most valued environmental ES in the Barcelona study, while placed in the worse ranking positions by Bologna citizens. Apart from these divergences, pollination was one of the most valued environmental ES in both systems. Overall, socio-cultural ES were the most valued among UA initiatives although the ranking of ES, particularly for environmental ES, could be influenced by the type of users (e.g., practitioners vs. citizens) and the local context.

Comparing the results presented herein with the evaluation of the ES provided by home gardens in rural areas [54], it was observed that recreation was also the most valued socio-cultural ES in home rural gardens. However, the participants from rural areas pointed to food production as the main ES of their home gardens, placing the provisioning services as the most important, in contrast to our results and those of Camps-Calvet et al. [40].

### Socio-demographic factors in future policy-making

The statistical assessment unveiled some socio-demographic factors that can be key for future policy-making. Social acceptance differences regarding gender, age and income level of the participant were significant for some of the aspects evaluated in this survey. In particular, gender, age and income can determine the preference for certain typologies of UA. Furthermore, gender and income level were also relevant in the evaluation of ES. These results suggested that UA planning shall include a participatory process where different citizens can provide inputs in the design and implementation of UA initiatives in Bologna, with the aim of encompassing these diverse preferences and perceived impacts of UA. On the other hand, the perception of risks and the type of activities that the participants would be willing to join showed also correlation with the socio-demographic characteristics. Thus, communication and awareness campaigns from local authorities aiming at affecting the social acceptance of UA may use these results to target certain population groups and topics.

### Study limits and generalizability

This study was based on a rigorous sampling approach. Nevertheless, by focusing on Bologna as a case study, the results were not representative of the entire population of Italy or even Southern Europe. It would be opportune to repeat the survey using a larger sample from all over Italy or even beyond, to include different local realities and socio-demographic differences. The repetition of the survey questionnaire, which was previously used in Berlin (Germany), allowed to draw some conclusions on common observations in both case studies. However, while the same questionnaire was applied in both cities, the sampling method in Berlin was based on a less structured, non-probability sampling approach and was characterized by a disproportionate number of urban, younger (20 to 40 years old) participants. This created a weakness with regards the comparability of the results. Nonetheless, both existing studies showed some common results and future studies on consumers’ preferences and acceptance can build on these results to generate hypotheses. As pointed out by Specht et al. [3], social acceptance is interrelated with local conditions and cultural values, which makes it necessary to compare results with other case studies in different geographical and cultural contexts.

## Conclusions

This paper contributes to an increased knowledge of the perception and acceptance of UA in Europe. We surveyed the inhabitants of Bologna, a landmark city in Southern Europe regarding the evolution and diversity of UA projects in the last decades. In particular, we evaluated the social acceptance in a comprehensive way, including the potential implementation of UA forms and the potential acceptance of the resulting products by identifying the preferences for urban land uses, UA typologies as well as UA products, investigating the perceived provision of ES by the citizens of Bologna and evaluating their willingness to engage in new UA initiatives. The outcomes resulted in the evaluation of new aspects of UA and with new data, which was compared and discussed with previous results in a broader European context.

The inhabitants of Bologna showed a wide acceptance of UA uses and products in their city, indicating that new initiatives might be positively received. While UA was conceptualized as multifunctional and diverse, animal production–including meadows -, rooftop agriculture and intensive farming were the least preferred. This result was in line with the low acceptance of intensive techniques and GMOs. As potential consumers, UA products were largely accepted although only a third of the population demonstrated a willingness-to-pay higher prices for UA products over conventional ones. Social ecosystem services were the most valued, as in other similar studies for urban and home gardens. The provision of food and medicinal plants were highlighted over the other environmental ES tied to UA.

Comparing Berlin and Bologna, specific trends regarding the acceptance of UA in Europe were observed. A diverse and multifunctional UA is appreciated in Europe, demonstrating the importance of specific UA policies for its development. The discourse of environmentally-friendly and resource efficiency was prevalent in both cities, although the citizens of Bologna also stressed the social nature and values of UA. Therefore, two main narratives were observed in European cities which could be addressed by policy-makers. In both cases, participants showed a broad acceptance of UA typologies and products, demonstrating the potential market success and diversification options of UA. Further research on other geographical areas might provide a more global vision of the acceptance of UA.

The results highlighted some policy-making recommendations for local authorities, which could be extended to similar European cities. First, more communication and dissemination activities regarding UA are needed, as the population of Bologna showed a lower awareness on the topic. Second, the citizens of Bologna showed a high willingness to engage in new UA projects, as both consumers and users. Thus, local authorities could promote not only the development of community-oriented UA activities but also the design of specific policies and norms for the development of UA businesses which will support entrepreneurs in overcoming current barriers and protect against perceived risks. Finally, the statistical assessment highlighted some differences between target groups which can set the basis for more precise policy-making regarding UA.

## Supporting information

S1 File Survey questionnaire. (DOC)

S2 File Summary of survey results. (DOC)

S3 File Survey results. (XLSX)

## Abbreviations

UA: Urban Agriculture
ES: Ecosystem Services
GMOs: genetically modified organisms
