# Supplementary material for: Social acceptance and perceived ecosystem services of urban agriculture in Southern Europe: The case of Bologna, Italy
Source: PLoS One. 2018 Sep 12;13(9):e0200993. doi: 10.1371/journal.pone.0200993 (PMC6135350; doi:10.1371/journal.pone.0200993)
Supplement: S1 File — (DOC) [file pone.0200993.s001.doc]

**SUPPLEMENTARY INFORMATION**

**Social Acceptance and Perceived Ecosystem Services of Urban Agriculture in Southern Europe: The Case of Bologna, Italy**

**Acceptance and Values of Urban Agriculture in Europe**

Esther Sanyé-Mengual^1^,*, Kathrin Specht^2^, Thomas Krikser^3^, Caterina Vanni^1^, Giuseppina Pennisi^1^, Francesco Orsini^1^, Giorgio Gianquinto^1^

*^1^Research Centre in Urban Environment for Agriculture and Biodiversity (ResCUE-AB), Department of Agricultural Sciences (Dipsa), Alma Mater Studiorium-University of Bologna, Viale Fanin 44, 40127, Bologna, Italy*

*^2^Department of Agricultural Economics, Humboldt-Universität zu Berlin, Unter den Linden 6, 10099, Berlin, Germany*

*^3^Department of Agricultural and Food Marketing, University of Kassel, Steinstrasse 19, 37213 Witzenhausen, Germany*

*Corresponding author:

E-mail: esther.sanye@unibo.it

**S1 FILE: SURVEY QUESTIONNAIRE**

| **Survey – Acceptance of urban agriculture in Bologna** |  |
| --- | --- |

**Organized by the University of Bologna we’re conducting a study on the acceptance of urban agriculture. To do so, we would like to ask you some questions. Would you be willing to participate?**

**(yes) Thanks for your cooperation**

**0a. Check if they comply with the conditions of the “target group”**

- live in Bologna?  Yes  No

- since more than 2 years?  Yes  No

- age: older than 18 years?  Yes  No

**0b. Ask for the neighborhood where they live:**

**-** City area (neighborhood):

**A. Urban agriculture**

1. If you think about your neighborhood, what would you say is generally missing?

Commercial areas (e.g., shopping center)

Educational areas (e.g., schools)

Residential areas (e.g., new buildings)

Industrial areas (e.g., new labor places)

Health services (e.g., hospitals)

Leisure areas (e.g., parks)

Public educational services (e.g., libraries, museums)

Financial services (e.g., banks)

Public administration services (e.g., administration offices)

Entertainment (e.g., cinema, theatres, bars)

Other______________________

1. Have you already hear about the term “urban agriculture”?  Yes  No
2. “Yes” answer: How would you explain/define it?

“No” answer: What do you think UA is like?

B. Types of urban agriculture (AU)

1. What of the following uses of green and open spaces would you like to have in your living surrounding? Valuation from 1 – strongly agree - to 5 – strongly disagree.

|  | Strongly agree |  |  |  | Strongly disagree |  |
| --- | --- | --- | --- | --- | --- | --- |
|  | 1 | 2 | 3 | 4 | 5 |  |
| **General preferences on urban land uses** |  |  |  |  |  |  |
| Public parks (for leisure and recreation) |  |  |  |  |  |  |
| Areas for nature protection and nature conservation |  |  |  |  |  |  |
| Demonstrative gardens (Maize- labyrinth, demonstration plots, educational trails) |  |  |  |  |  |  |
| Meadows (for sheep, cattle, horses, donkeys) |  |  |  |  |  |  |
| Public accessible intercultural gardens (volunteer initiatives) |  |  |  |  |  |  |
| Residential gardens (rental plots for inhabitants) |  |  |  |  |  |  |
| **Preferences for different UA types**  **(Show pics – visual help)** |  |  |  |  |  | I don’t know |
| Agricultural farms (intensive agricultural and horticultural landscapes) |  |  |  |  |  |  |
| UA in the urban periphery |  |  |  |  |  |  |
| UA in agricultural parks in the periphery |  |  |  |  |  |  |
| UA in Pick-your-own projects |  |  |  |  |  |  |
| UA on inner-city brownfields |  |  |  |  |  |  |
| UA in public parks |  |  |  |  |  |  |
| UA in mobile modules (all around the city) |  |  |  |  |  |  |
| UA in backyards (for residents or groups of residents) |  |  |  |  |  |  |
| Private rooftop gardens |  |  |  |  |  |  |
| Rooftop farms (commercial) |  |  |  |  |  |  |
| UA in rooftop greenhouses |  |  |  |  |  |  |
| UA in aquaponic farms (where fish and vegetables are produced) |  |  |  |  |  |  |
| UA in vertical farms (multi-story buildings where the building is 100% devoted to food production) |  |  |  |  |  |  |
| Social UA for groups in social exclusion risk (migrants, youth) |  |  |  |  |  |  |

C. UA production systems

1. Now we speak about different production systems that can be used in UA. How is your attitude towards the following production systems and orientations? Valuation from 1 – strongly approve - to 5 – strongly reject.

|  | Strongly approve |  |  |  | Strongly reject |
| --- | --- | --- | --- | --- | --- |
|  | 1 | 2 | 3 | 4 | 5 |
| Use of: |  |  |  |  |  |
| - Greenhouse |  |  |  |  |  |
| - Soil-less production |  |  |  |  |  |
| - MGOs |  |  |  |  |  |
| Production of vegetables: |  |  |  |  |  |
| - Resource oriented organic farming (use of rain water, waste heat, renewable energy) |  |  |  |  |  |
| - Organic farming (pesticides and fertilizer) |  |  |  |  |  |
| - Extensive |  |  |  |  |  |
| - Intensive |  |  |  |  |  |
| Intensive production of livestock |  |  |  |  |  |

D. UA products

6. Now, I will name diverse products that can be produced in cities. Could you give us your opinion regarding:

- What products do you know (seen or listen about) can be produced in Bologna?

- What products would you approve to be produced through UA in Bologna?

- What products would you be willing to buy from UA in Bologna?

| Possible UA products | It’s produced in Bologna | I would approve their production in Bologna | I would buy from UA in Bologna |
| --- | --- | --- | --- |
| Vegetables, open ground |  |  |  |
| Vegetables, greenhouse |  |  |  |
| Fruits and wine, orchards |  |  |  |
| livestock products  milk/cheese  honey  wool  eggs  meat |  |  |  |
| Arable crops (wheat, potatoes) |  |  |  |
| Specialty products (mushrooms, sprouts, herbs) |  |  |  |
| Aquaponic (fish and vegetables) |  |  |  |

1. What aspects would make you buy UA products instead of products from conventional agriculture? (Choose the most important factor for your decision)

distance: local production

production type: organic production

higher quality (e.g., freshness, nutritional value, aspect)

complementary social characteristics of UA projects (e.g., educational programs for children)

certified products (food safety)

other __________________________________________________

1. Now we value the willingness to pay. When 1kg of onions from conventional and imported agriculture costs 1€ in the supermarket, how would you be willing to pay for 1kg of onions from UA produced and sold in Bologna?

I don’t know

the same value

other value: ____

1. Considering now the different risks associated to UA. What is your opinion related to these impacts, according to the following statements? Valuation from 1 – strongly agree - to 5 – strongly disagree.

|  | Strongly agree |  |  |  | Strongly disagree |
| --- | --- | --- | --- | --- | --- |
|  | 1 | 2 | 3 | 4 | 5 |
| Risk of pollution by: |  |  |  |  |  |
| - Air contamination (industries, mobility) |  |  |  |  |  |
| - Soil contamination |  |  |  |  |  |
| Soil-less growing is an ‘‘unnatural’’ way of producing |  |  |  |  |  |
| Competition with: |  |  |  |  |  |
| - Rural farmers |  |  |  |  |  |
| - other uses (like solar energy on the roof, or spaces in the city for parks, residential areas etc.) |  |  |  |  |  |
| Leads to non- appropriate animal keeping |  |  |  |  |  |
| Leads to increased noise, dirt and smell due to: |  |  |  |  |  |
| - vegetable production |  |  |  |  |  |
| - animal production |  |  |  |  |  |

E. Ecosystem services

1. Please, value the different ecosystem services associated to urban food systems by answering the following statement: “Urban food systems contribute to…”. Value scale from 1 – strongly agree - to 5 –strongly disagree.

| ENVIRONMENTAL SERVICES | Strongly agree |  |  |  | Strongly disagree |
| --- | --- | --- | --- | --- | --- |
|  | 1 | 2 | 3 | 4 | 5 |
| **Provision of:** |  |  |  |  |  |
| - Food |  |  |  |  |  |
| - Medicine and aromatic plants |  |  |  |  |  |
| - Other raw materials (e.g. wool) |  |  |  |  |  |
| **Regulation services** |  |  |  |  |  |
| Improvement of |  |  |  |  |  |
| - Local climate |  |  |  |  |  |
| - Air quality |  |  |  |  |  |
| Facilitation of |  |  |  |  |  |
| - Carbon sequestration and storage |  |  |  |  |  |
| - Pollination |  |  |  |  |  |
| - Biological pest control |  |  |  |  |  |
| Reduction of the effects of extreme events (e.g., flood, hurricane) |  |  |  |  |  |
| Prevention of soil erosion and maintenance of soil fertility |  |  |  |  |  |
| Regulation of the urban metabolism (e.g., waste, water and nutrient flows) |  |  |  |  |  |
| **Habitat** |  |  |  |  |  |
| Habitat for animal species |  |  |  |  |  |
| Conservation of genetic variability |  |  |  |  |  |
| Increase of global biodiversity |  |  |  |  |  |

F. Society and surrounding

1. Please, value the different ecosystem services associated to urban food systems by answering the following statement: “Urban food systems contribute to…”

Value scale from 1 – strongly agree - to 5 –strongly disagree.

| SOCIO-CULTURAL SERVICES | Strongly agree |  |  |  | Strongly disagree |
| --- | --- | --- | --- | --- | --- |
|  | 1 | 2 | 3 | 4 | 5 |
| Education and training |  |  |  |  |  |
| Recreation and entertainment |  |  |  |  |  |
| Improvement of urban tourist attraction |  |  |  |  |  |
| Improvement of |  |  |  |  |  |
| - Mental health (relax, stress) |  |  |  |  |  |
| - Physical health |  |  |  |  |  |
| - Contact with nature and spiritual experiences |  |  |  |  |  |
| Urban aesthetics and cultural/art inspiration |  |  |  |  |  |
| Maintenance of traditional knowledge and cultural heritage (e.g., local varieties, production techniques, etc.) |  |  |  |  |  |
| Improvement of:   - Community building |  |  |  |  |  |
| - Social cohesion |  |  |  |  |  |
| - Place attachment |  |  |  |  |  |
| Contribution to political realization |  |  |  |  |  |

1. Do you think that UA can improve the image of your city?

large improvement

improvement

without changes

deterioration

I don’t know

G. Participation

1. Could you imagine to participate in UA? How? (multiple entries possible)

Own production of products

Buying products

Distributing products

providing financial support

attending educational and training programs

using the space for relaxation

organizing events

other___________________________________

I won’t participate

1. In which area would you participate or contribute in an UA project?

in the entire city

in my own neighborhood

I won’t participate

Statistical data

Now we ask for some information needed for the statistical assessment:

1. Sex:  male  female
2. Age:  18-29  30-44  45-64  65-79  >80
3. Household income (monthly):

≤ 1000  1001-3000  3001-5000  > 5000

1. Number of people living at your household unit: __

**Comments**

VISUAL AID – Question 4b

| 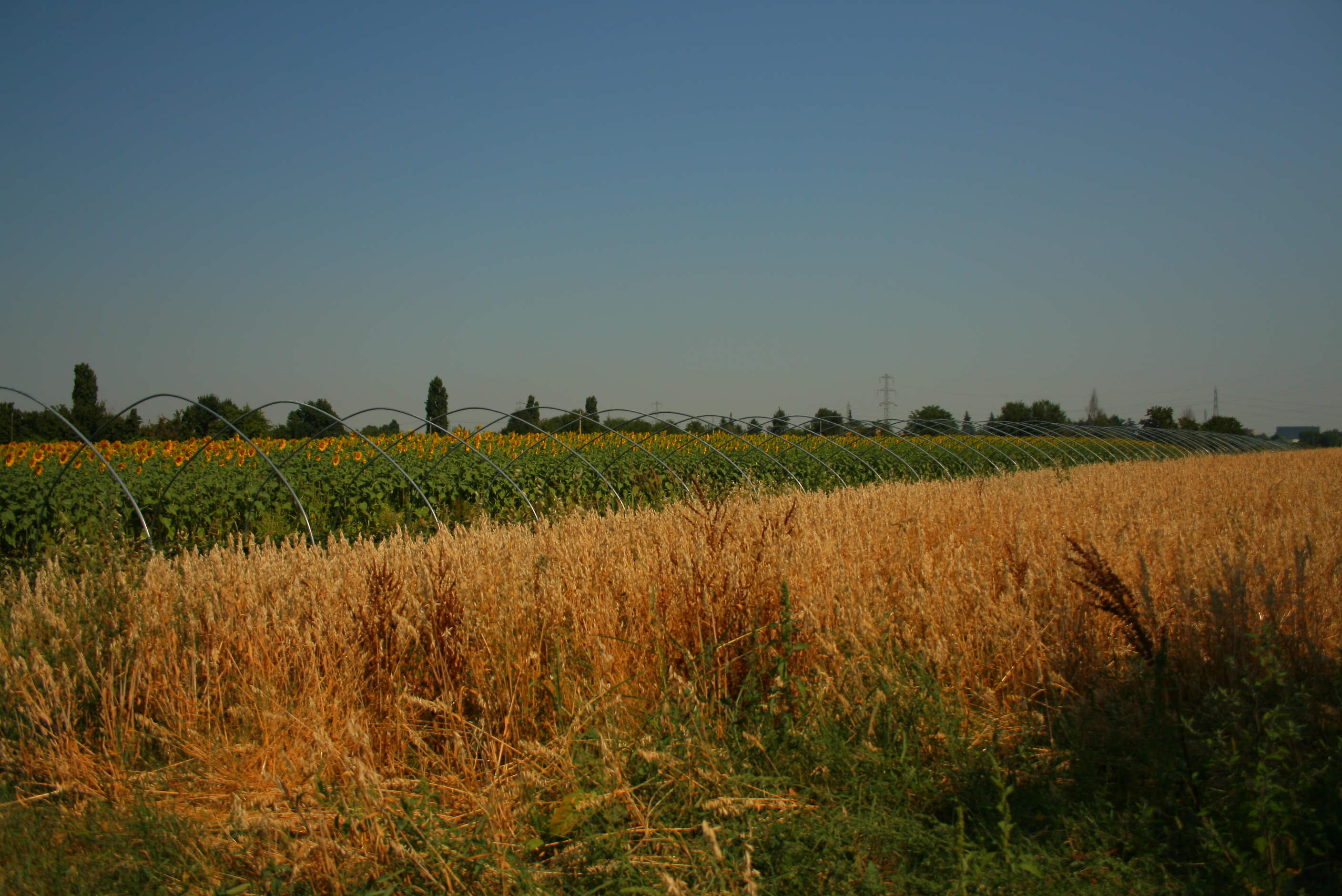 | 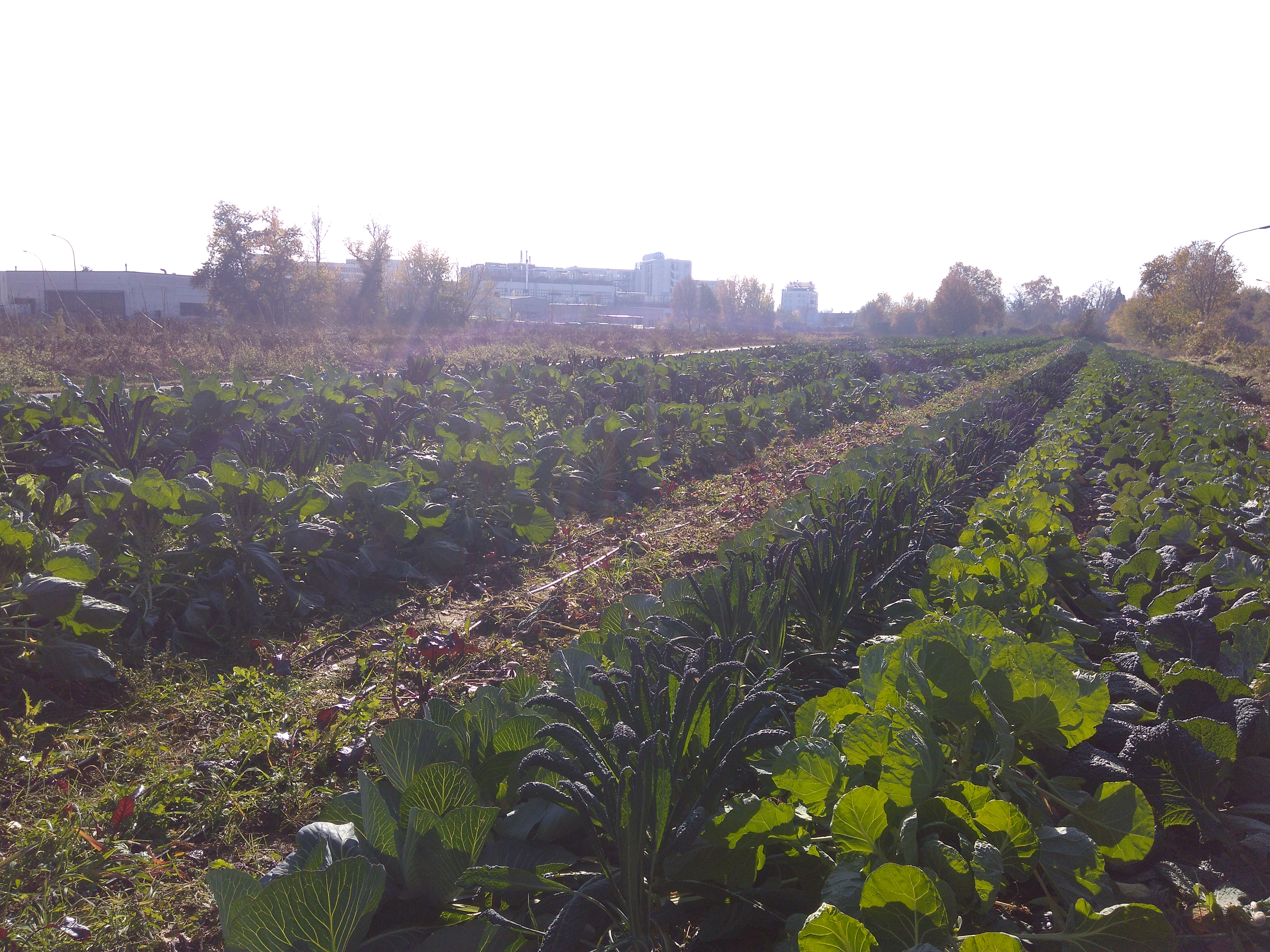 |
| --- | --- |
| Agricultural farms (intensive agricultural and horticultural landscapes) | UA in the urban periphery |
| 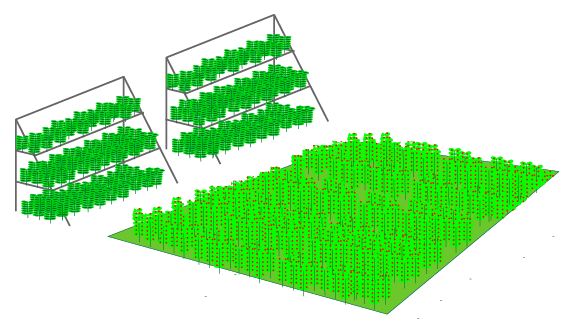 | 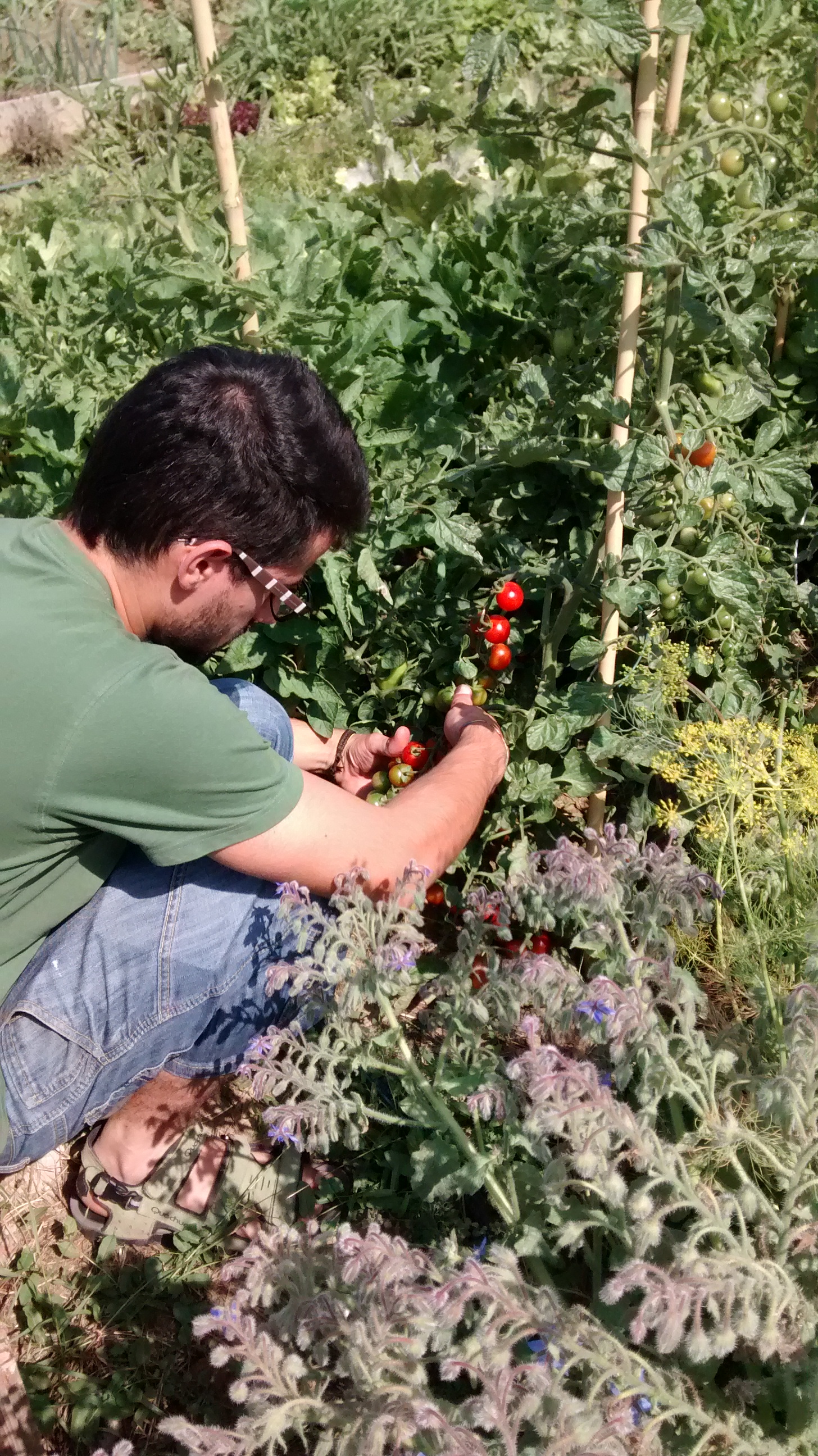 |
| UA in agricultural parks in the periphery | UA in Pick-your-own projects |
| 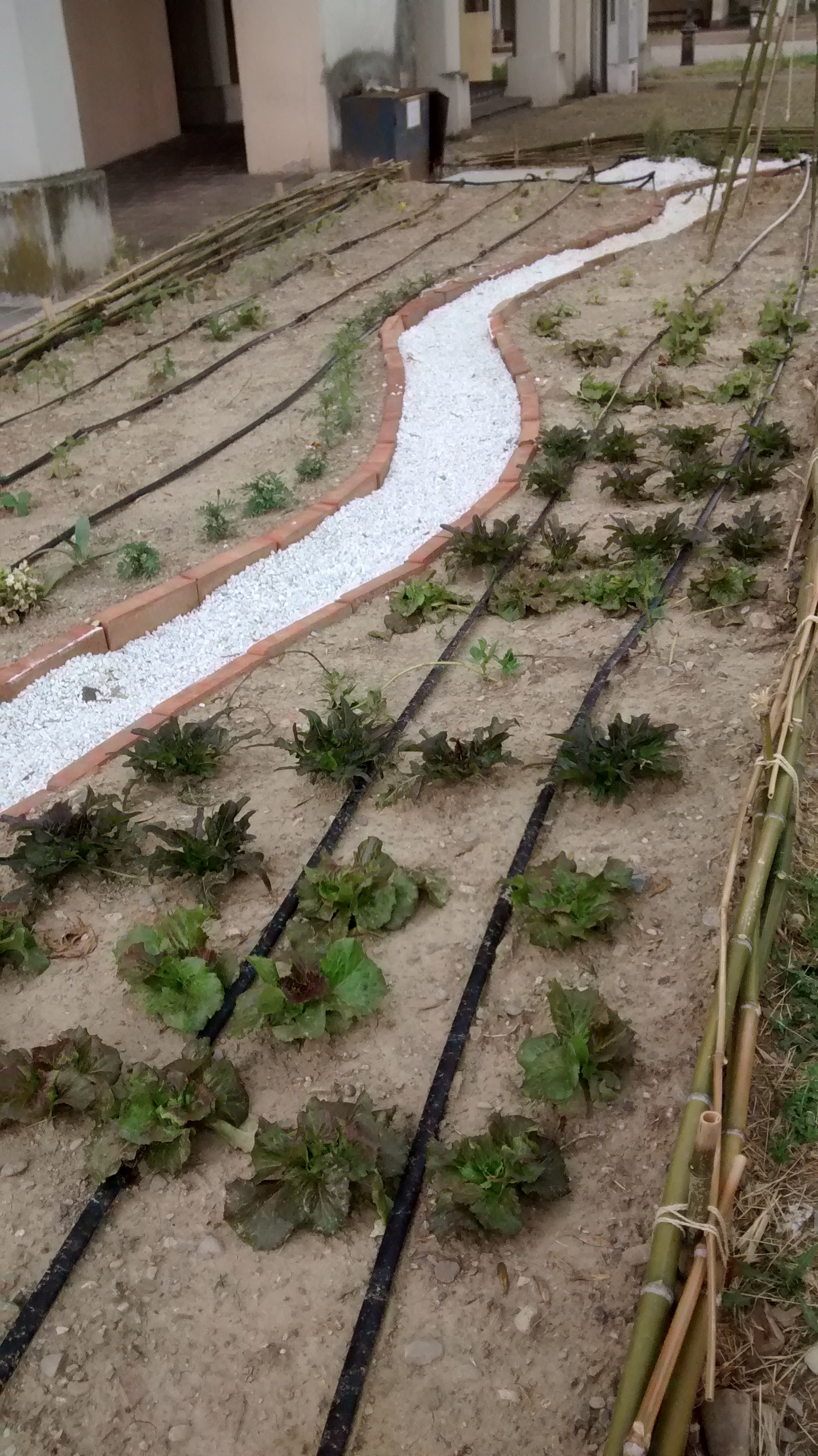 | 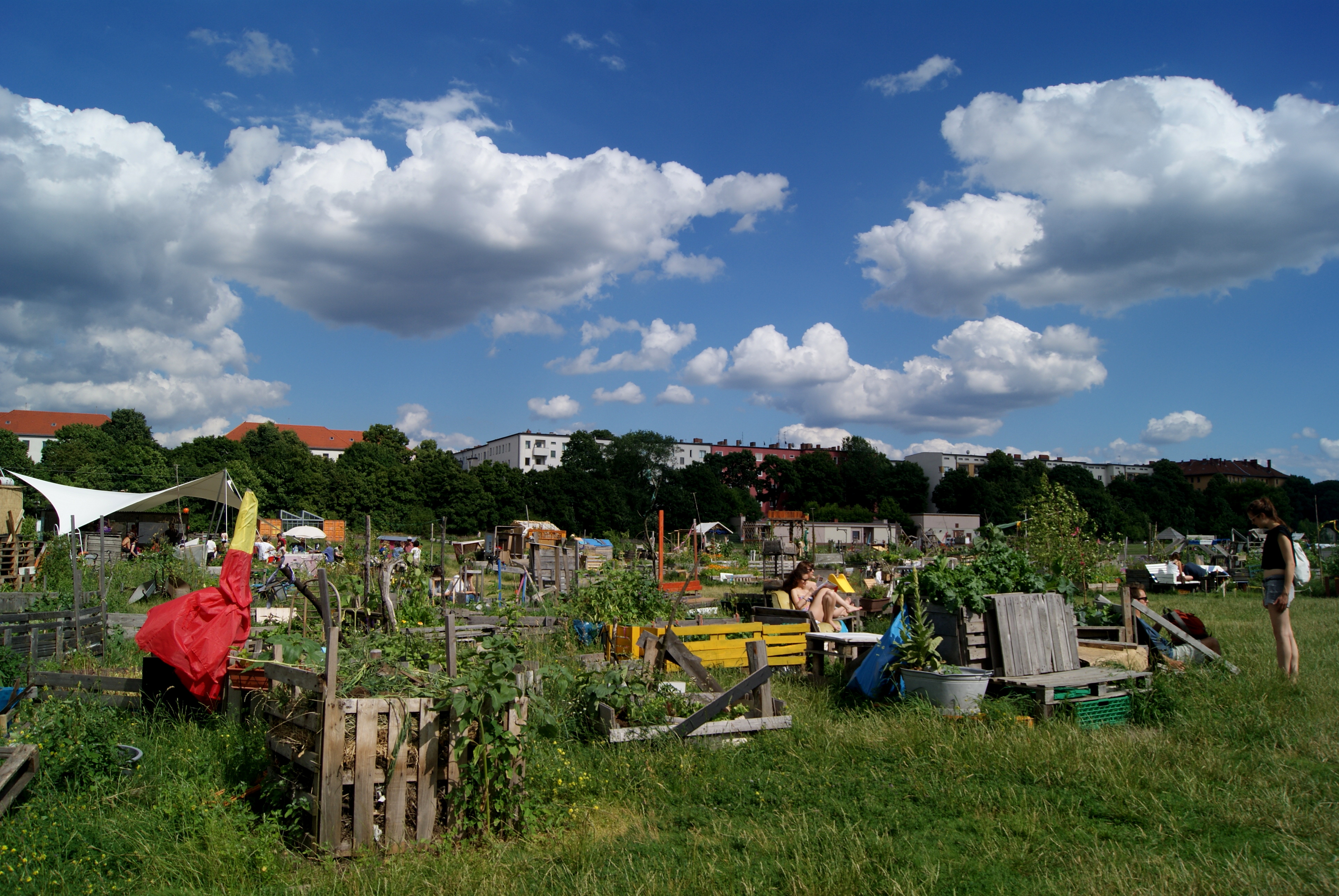 |
| UA on inner-city brownfields | UA in public parks |
| 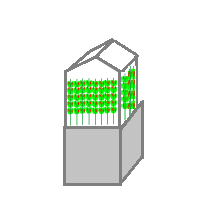 | 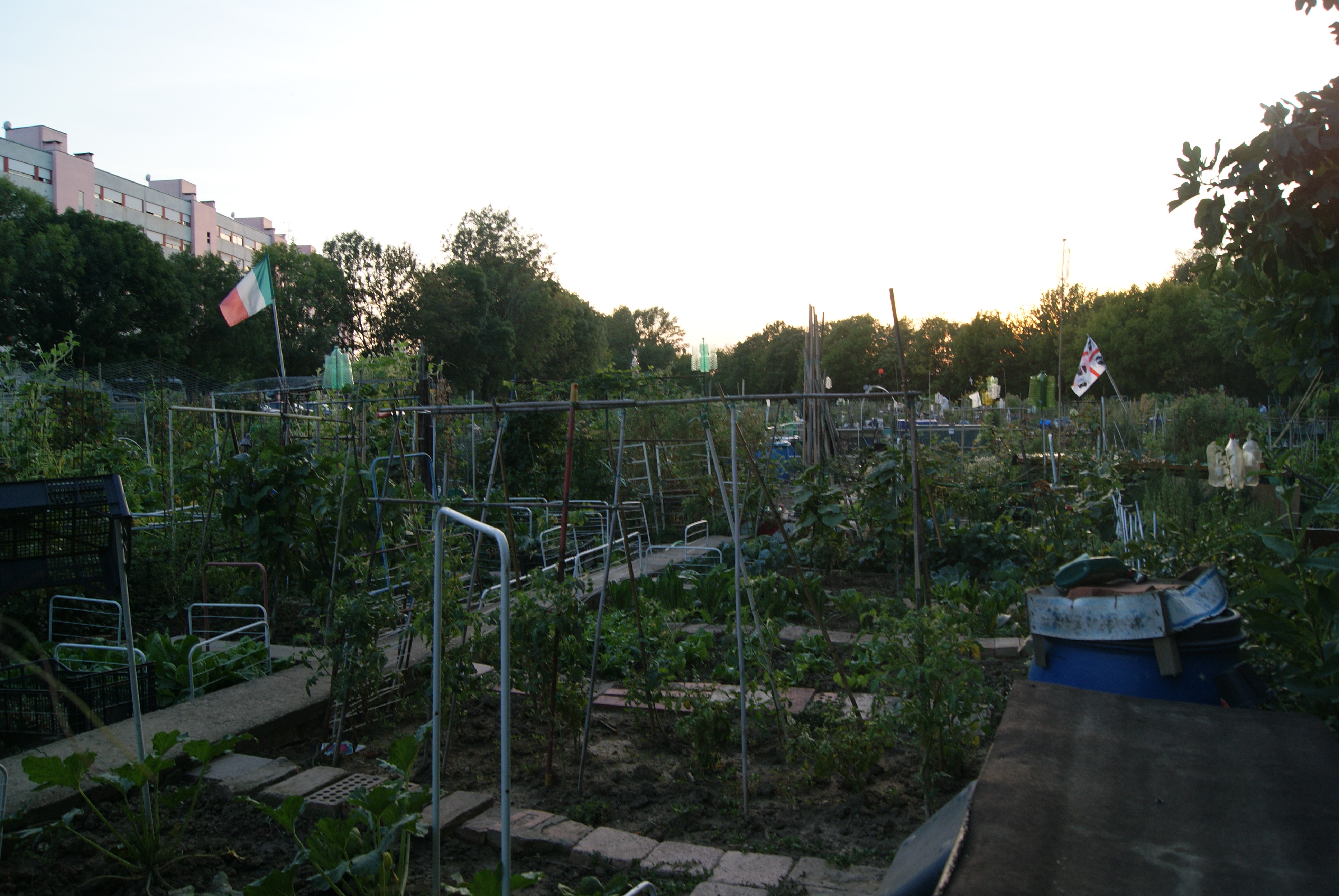 |
| UA in mobile modules (all around the city) | UA in backyards (for residents or groups of residents) |
| 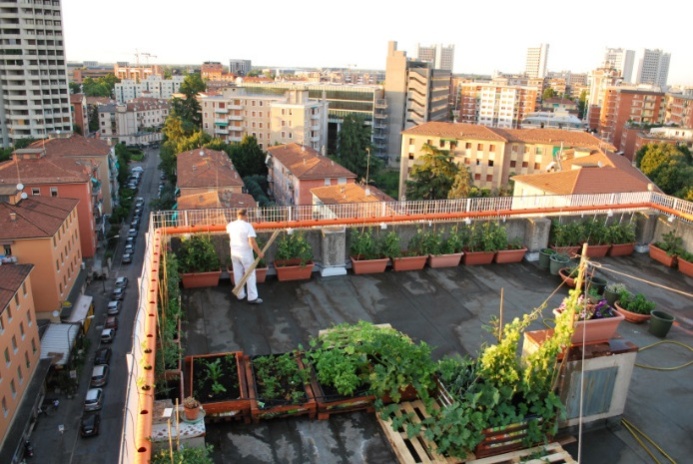 | 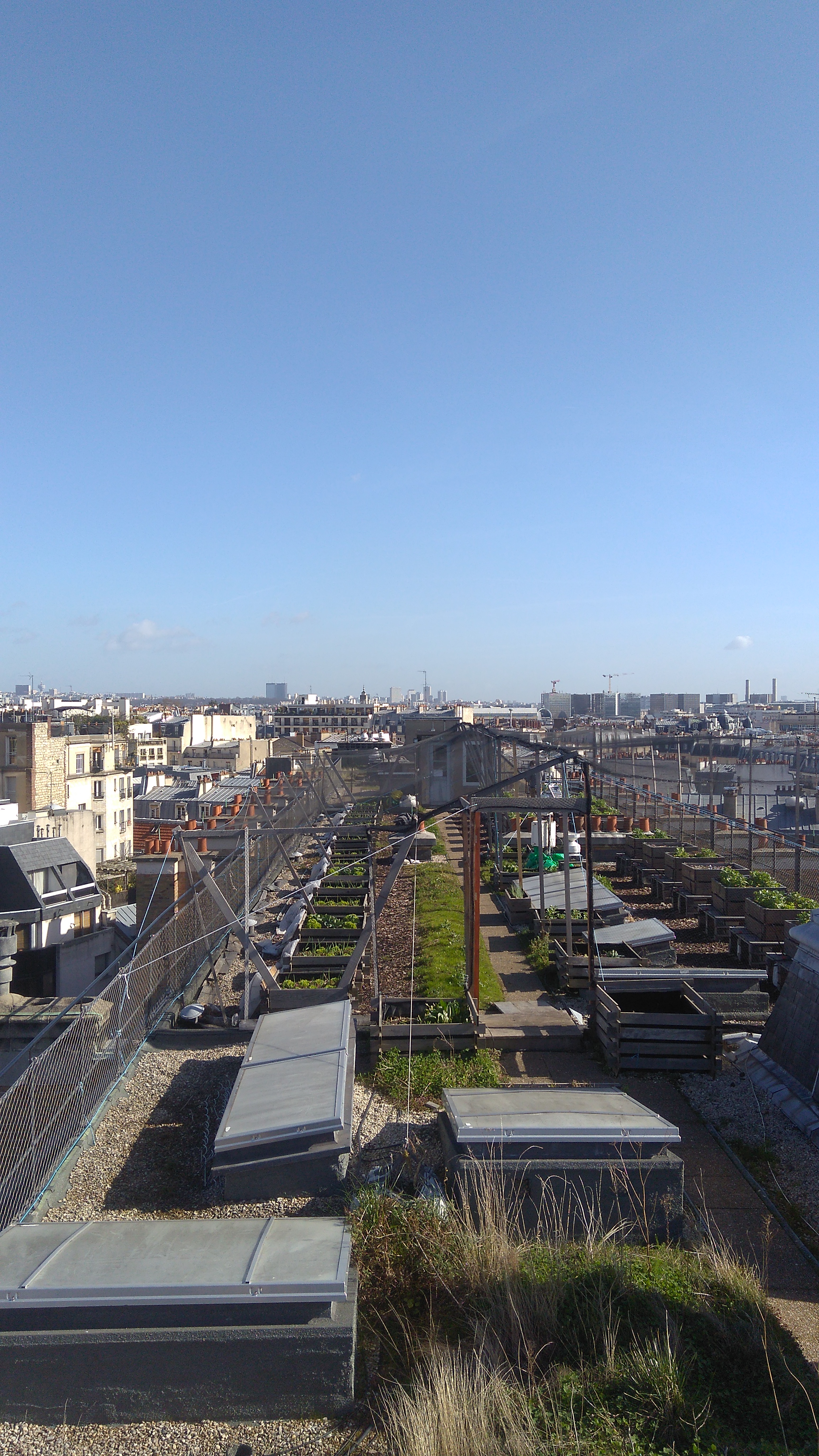 |
| Private rooftop gardens | Rooftop farms (commercial) |
| 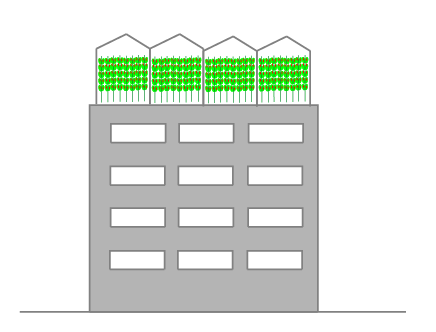 | 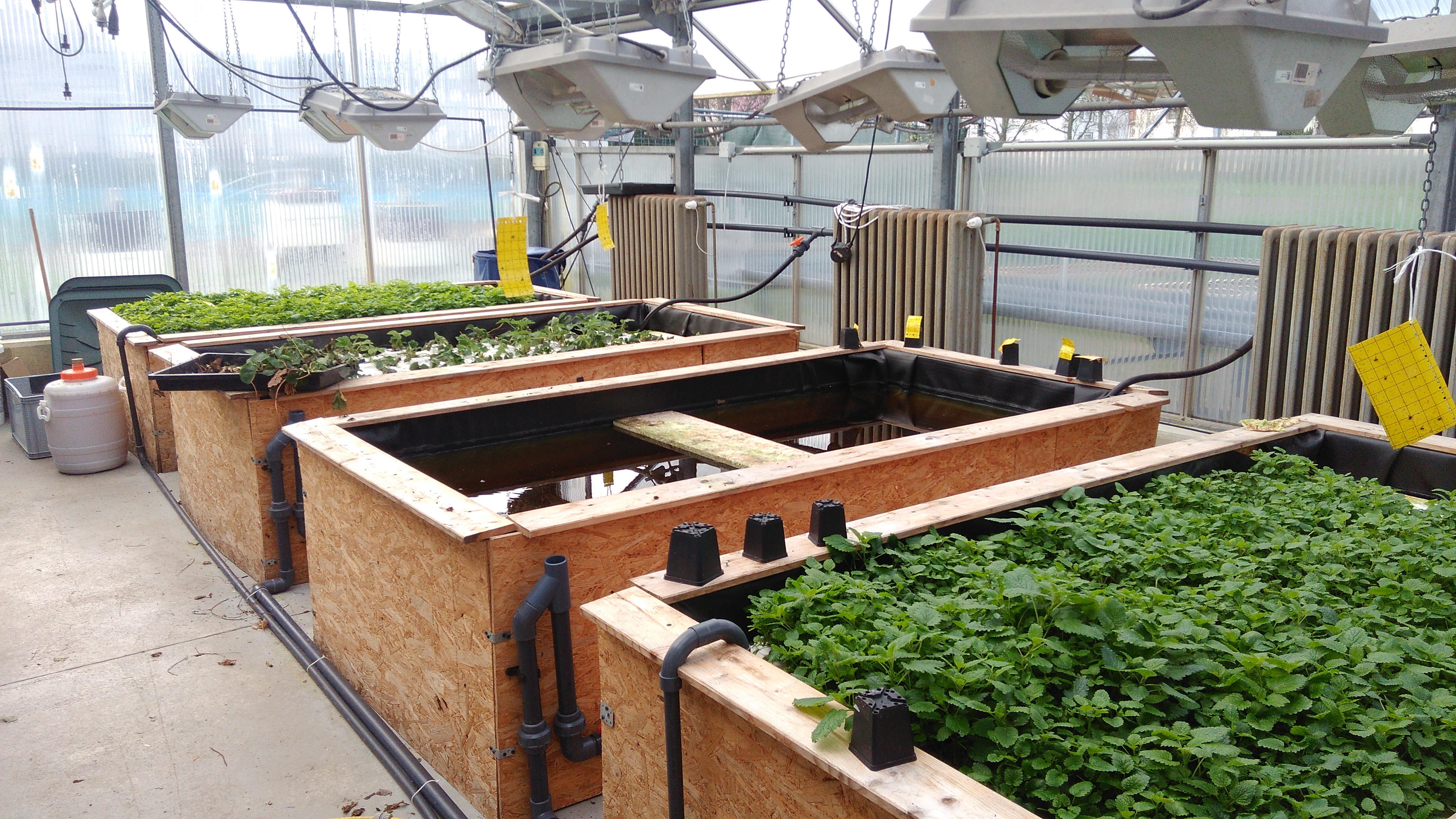  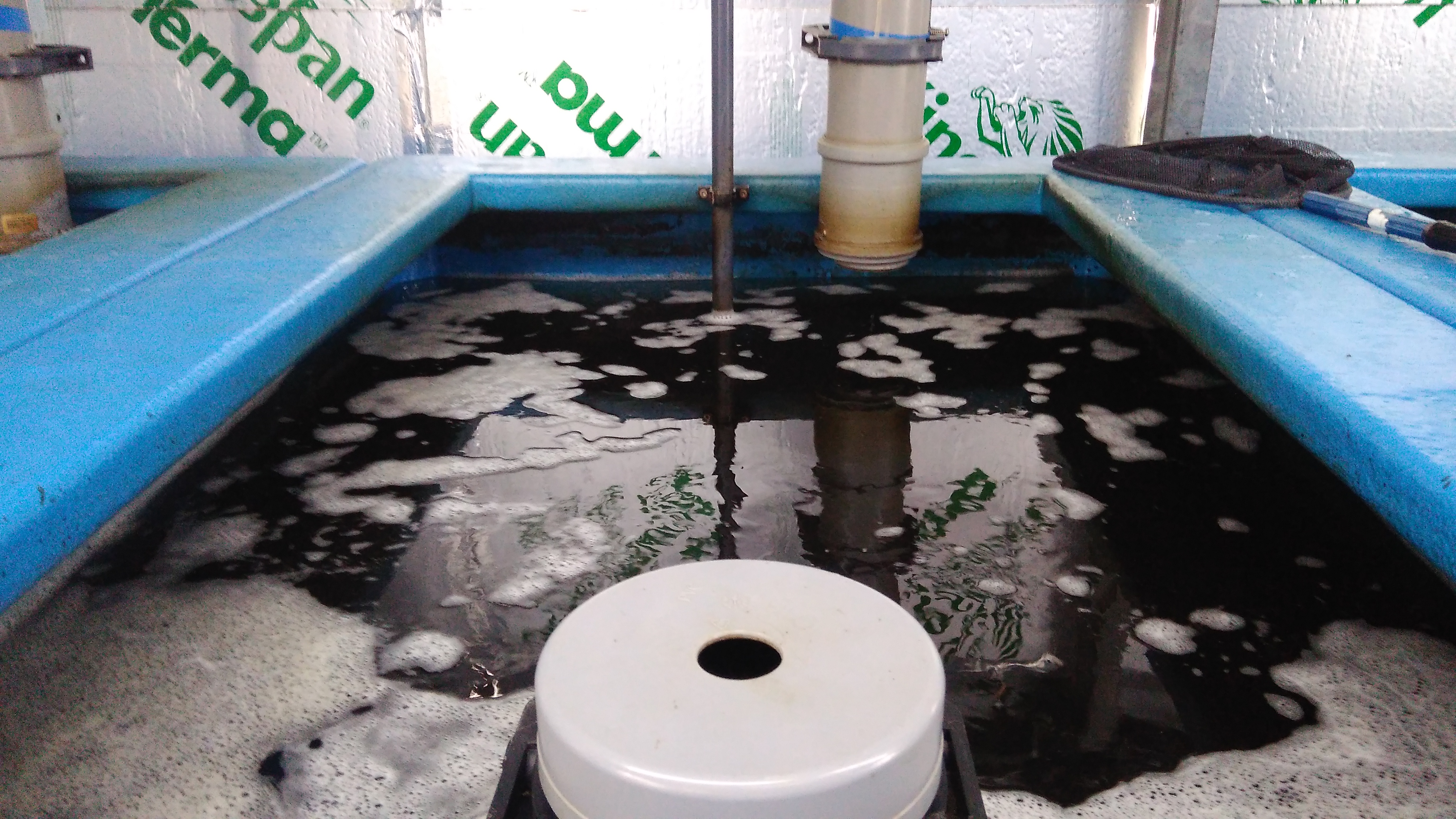 |
| UA in rooftop greenhouses | UA in aquaponic farms (where fish and vegetables are produced) |
| 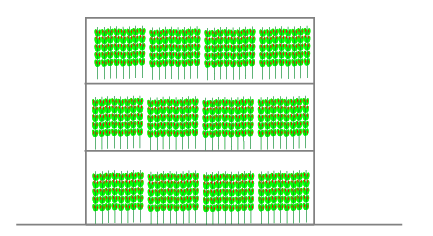 | 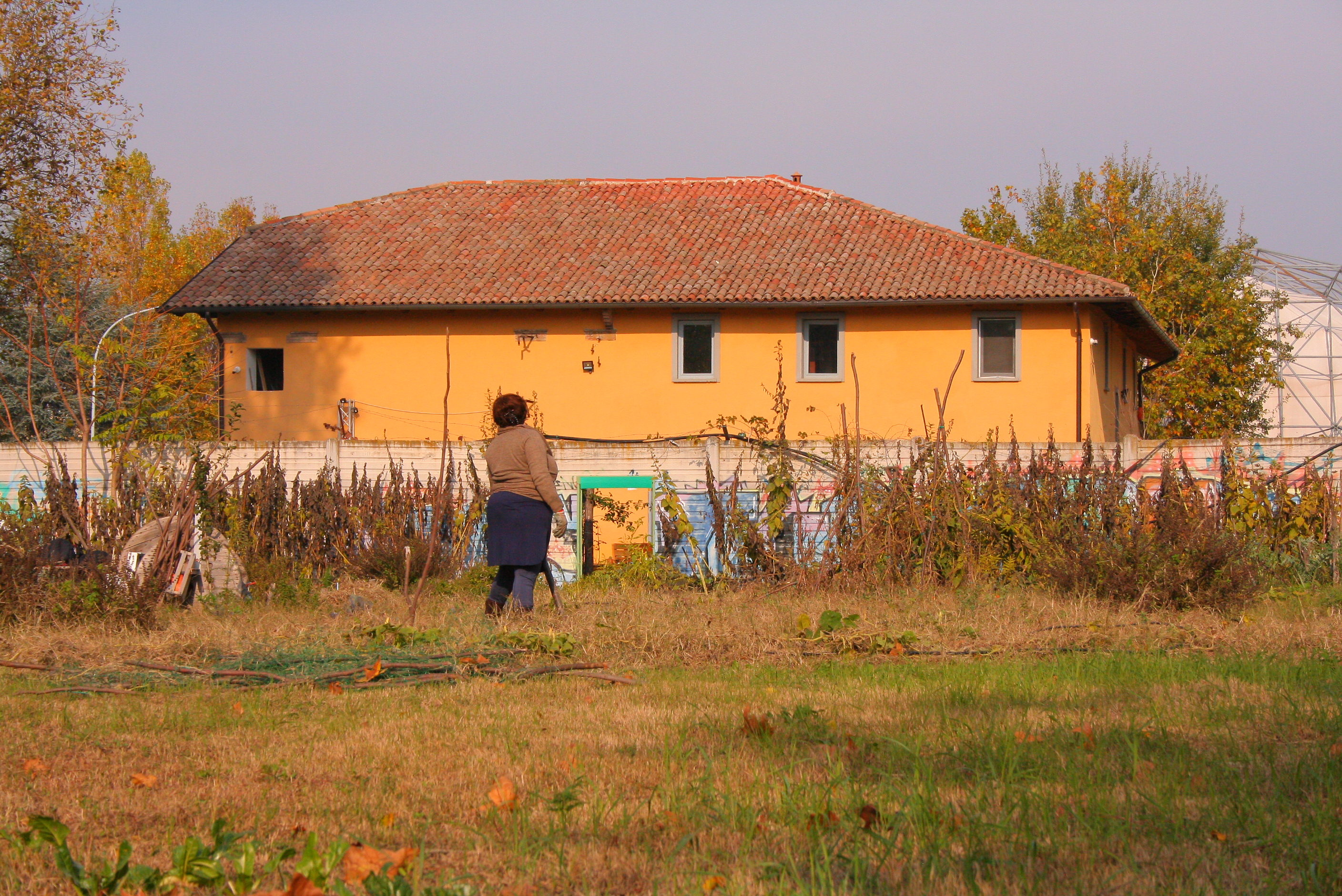 |
| UA in vertical farms (multi-storey buildings where the building is 100% devoted to food production) | Social UA for groups in social exclusion risk (migrants, youth) |

*The figures used in this version of the survey are similar but not identical to the original images used in the study, and they are therefore for illustrative purposes only.
