# Supplementary material for: Social acceptance and perceived ecosystem services of urban agriculture in Southern Europe: The case of Bologna, Italy
Source: PLoS One. 2018 Sep 12;13(9):e0200993. doi: 10.1371/journal.pone.0200993 (PMC6135350; doi:10.1371/journal.pone.0200993)
Supplement: S2 File — (DOC) [file pone.0200993.s002.doc]

**SUPPLEMENTARY INFORMATION**

**Social Acceptance and Perceived Ecosystem Services of Urban Agriculture in Southern Europe: The Case of Bologna, Italy**

**Acceptance and Values of Urban Agriculture in Europe**

Esther Sanyé-Mengual^1^,*, Kathrin Specht^2^, Thomas Krikser^3^, Caterina Vanni^1^, Giuseppina Pennisi^1^, Francesco Orsini^1^, Giorgio Gianquinto^1^

*^1^Research Centre in Urban Environment for Agriculture and Biodiversity (ResCUE-AB), Department of Agricultural Sciences (Dipsa), Alma Mater Studiorium-University of Bologna, Viale Fanin 44, 40127, Bologna, Italy*

*^2^Department of Agricultural Economics, Humboldt-Universität zu Berlin, Unter den Linden 6, 10099, Berlin, Germany*

*^3^Department of Agricultural and Food Marketing, University of Kassel, Steinstrasse 19, 37213 Witzenhausen, Germany*

*Corresponding author:

E-mail: esther.sanye@unibo.it

**S2 FILE: SURVEY RESULTS**

This supplementary information presents the raw results of the survey, by section and question (Answers to the open question #3 have not been included).

**A. Urban agriculture**

1. If you think about your neighborhood, what would you say is generally missing? (n=380)

|  | **Yes (n)** | **No (n)** | **NA (n)** | **Total (n)** |
| --- | --- | --- | --- | --- |
| **Financial services** | 12 | 234 | 134 | 380 |
| **Residential areas** | 17 | 230 | 133 | 380 |
| **Administrative services** | 24 | 229 | 127 | 380 |
| **Industrial areas** | 35 | 220 | 125 | 380 |
| **Health servicces** | 55 | 196 | 129 | 380 |
| **Educational services** | 68 | 182 | 130 | 380 |
| **Commercial services** | 76 | 190 | 114 | 380 |
| **Recreational areas** | 192 | 98 | 90 | 380 |
| **Public cultural services** | 199 | 106 | 75 | 380 |
| **Entertainment** | 204 | 121 | 55 | 380 |

1. Have you already hear about the term “urban agriculture”?

|  | **Yes (n)** | **No (n)** | **NA (n)** | **Total (n)** |
| --- | --- | --- | --- | --- |
| **Respondents** | 175 | 201 | 2 | 380 |

1. “Yes” answer: How would you explain/define it?

“No” answer: What do you think UA is like?

[Open answers not included]

B. Types of urban agriculture (AU)

1. What of the following uses of green and open spaces would you like to have in your living surrounding? Valuation from 1 – strongly agree - to 5 – strongly disagree.

Part A: Uses of green spaces

|  | **1** | **2** | **3** | **4** | **5** | **(NA)** | **Total (n)** |
| --- | --- | --- | --- | --- | --- | --- | --- |
| **Meadows**  **(n=367; avg=3,29)** | 62 | 51 | 69 | 88 | 97 | 13 | 367 |
| **Demonstrative gardens**  **(n=378; avg=1,40)** | 259 | 93 | 21 | 4 | 1 | 2 | 378 |
| **Intercultural gardens accessible to the public**  **(n=378; avg=1,36)** | 282 | 70 | 17 | 5 | 4 | 2 | 378 |
| **Natural protection areas**  **(n=379; avg=1,30)** | 285 | 81 | 8 | 3 | 2 | 1 | 379 |
| **Gardens for residents**  **(n=383; avg=1,30)** | 292 | 68 | 12 | 7 | 0 | 1 | 383 |
| **Public parks**  **(n=377; avg=1,15)** | 325 | 49 | 0 | 3 | 0 | 3 | 377 |

Part B: Typologies of urban agriculture

|  | **1** | **2** | **3** | **4** | **5** | **DK** | **NA** | **Total** |
| --- | --- | --- | --- | --- | --- | --- | --- | --- |
| **Intensive agriculture**  **(n=378; avg=2.14)** | 177 | 74 | 62 | 25 | 39 | 1 | 2 | 378 |
| **Rooftop farms**  **(n=378; avg=1.92)** | 196 | 88 | 34 | 28 | 25 | 7 | 2 | 378 |
| **Private rooftop gardens**  **(n=379; avg=1.57)** | 236 | 93 | 33 | 9 | 7 | 1 | 1 | 379 |
| **Rooftop greenhouses**  **(n=378; avg=1.56)** | 246 | 78 | 28 | 11 | 11 | 4 | 2 | 378 |
| **Gardens in public parks**  **(n=378; avg=1.56)** | 241 | 92 | 21 | 14 | 9 | 1 | 2 | 378 |
| **Container farms around the city**  **(n=379; avg=1.56)** | 234 | 95 | 23 | 10 | 9 | 8 | 1 | 379 |
| **Acquaponics**  **(n=377; avg=1.51)** | 259 | 64 | 22 | 11 | 12 | 9 | 3 | 377 |
| **Vertical farming**  **(n=377; avg=1.51)** | 245 | 81 | 19 | 10 | 9 | 13 | 3 | 377 |
| **Private gardens**  **(n=377; avg=1.50)** | 241 | 95 | 26 | 8 | 4 | 3 | 3 | 377 |
| **Pick-Your-Own gardens**  **(n=379; avg=1.39)** | 272 | 74 | 22 | 5 | 3 | 3 | 1 | 379 |
| **Periurban parks**  **(n=380; avg=1.38)** | 264 | 95 | 14 | 3 | 3 | 1 | 0 | 380 |
| **Periurban agriculture**  **(n=378; avg=136)** | 269 | 89 | 14 | 4 | 2 | 0 | 2 | 378 |
| **Gardens for social inclusion**  **(n=378; avg=1.34)** | 295 | 49 | 14 | 5 | 9 | 6 | 2 | 378 |
| **Gardens on vacant lands**  **(n=377; avg=1.34)** | 275 | 83 | 13 | 2 | 3 | 1 | 3 | 377 |

C. UA production systems

1. Now we speak about different production systems that can be used in UA. How is your attitude towards the following production systems and orientations? Valuation from 1 – strongly approve - to 5 – strongly reject.

|  | **1** | **2** | **3** | **4** | **5** | **DK** | **NA** | **Total** |
| --- | --- | --- | --- | --- | --- | --- | --- | --- |
| **Intensive production of animals (n=382)** | 18 | 49 | 77 | 71 | 163 | 0 | 2 | 380 |
| **Use of GMOs (n=382)** | 37 | 55 | 91 | 91 | 104 | 0 | 2 | 380 |
| **Intensive production of vegetables (n=380)** | 93 | 85 | 77 | 42 | 79 | 0 | 4 | 380 |
| **Extensive production (n=381)** | 170 | 91 | 58 | 35 | 23 | 0 | 3 | 380 |
| **Soil-less production (n=383)** | 232 | 105 | 28 | 11 | 3 | 0 | 1 | 380 |
| **Greenhouse technology (n=383)** | 251 | 105 | 18 | 3 | 2 | 0 | 1 | 380 |
| **Biological production (n=383)** | 317 | 51 | 9 | 2 | 0 | 0 | 1 | 380 |
| **Resource-efficient agriculture (n=380)** | 313 | 55 | 8 | 1 | 0 | 0 | 3 | 380 |

D. UA products

6. Now, I will name diverse products that can be produced in cities. Could you give us your opinion regarding:

- What products do you know (seen or listen about) can be produced in Bologna?

- What products would you approve to be produced through UA in Bologna?

- What products would you be willing to buy from UA in Bologna?

|  | **I would buy (%)** | **n** | **I would accept (%)** | **n** | **I know (%)** | **n** |
| --- | --- | --- | --- | --- | --- | --- |
| **Seeds** | 92,6 | 171 | 94,8 | 253 | 93,9 | 230 |
| **Specialties** | 91,9 | 180 | 92,4 | 288 | 82,3 | 195 |
| **Fruit** | 91,6 | 175 | 93,6 | 238 | 90,9 | 241 |
| **Greenhouse products** | 89,8 | 163 | 97,6 | 251 | 95,3 | 253 |
| **Aquaponics** | 86,8 | 186 | 90,6 | 303 | 61,9 | 160 |
| **Honey** | 86,5 | 188 | 89,7 | 283 | 79,1 | 173 |
| **Vegetables** | 83,4 | 165 | 90,2 | 249 | 97,4 | 247 |
| **Egg** | 76,5 | 177 | 82,1 | 249 | 82,1 | 195 |
| **Milk** | 73,0 | 176 | 77,9 | 235 | 92,8 | 219 |
| **Whool** | 70,1 | 171 | 81,5 | 300 | 52,5 | 148 |
| **Meat** | 69,4 | 177 | 76,0 | 246 | 78,8 | 197 |

1. What aspects would make you buy UA products instead of products from conventional agriculture? (Choose the most important factor for your decision)

| **Motivation** | **Respondents (n)** |
| --- | --- |
| **Social functions** | 93 |
| **Distance** | 81 |
| **Quality** | 73 |
| **Type of production** | 70 |
| **Certification of products** | 56 |
| **Other** | 4 |
| **Price** | 2 |
| **Total** | 379 |

1. Now we value the willingness to pay. When 1kg of onions from conventional and imported agriculture costs 1€ in the supermarket, how would you be willing to pay for 1kg of onions from UA produced and sold in Bologna?

| **Value (€)** | **Respondents (n)** |
| --- | --- |
| **0,9** | 1 |
| **0,95** | 1 |
| **1** | 182 |
| **1,05** | 5 |
| **1,1** | 7 |
| **1,2** | 16 |
| **1,3** | 11 |
| **1,35** | 1 |
| **1,5** | 17 |
| **1,8** | 5 |
| **2** | 11 |
| **2,5** | 2 |
| **3** | 4 |
| **Total** | **263** |

1. Considering now the different risks associated to UA. What is your opinion related to these impacts, according to the following statements? Valuation from 1 – strongly agree - to 5 – strongly disagree.

|  | **1** | **2** | **3** | **4** | **5** | **DK** | **NA** | **Total** |
| --- | --- | --- | --- | --- | --- | --- | --- | --- |
| **Soil-less is unnatural** | 29 | 59 | 115 | 103 | 71 | 0 | 3 | 377 |
| **Air contamination** | 134 | 117 | 59 | 43 | 26 | 0 | 1 | 379 |
| **Soil contamination** | 138 | 109 | 64 | 44 | 24 | 0 | 1 | 379 |
| **Animal management** | 68 | 68 | 100 | 75 | 66 | 0 | 3 | 377 |
| **Noise from vegetable production** | 70 | 60 | 118 | 76 | 54 | 0 | 2 | 378 |
| **Noise from animal production** | 91 | 78 | 100 | 60 | 47 | 0 | 4 | 376 |
| **Competition with conventional agriculture** | 68 | 142 | 65 | 65 | 35 | 0 | 5 | 375 |
| **Competition with other uses** | 97 | 117 | 79 | 57 | 29 | 0 | 1 | 379 |

E. Ecosystem services

1. Please, value the different ecosystem services associated to urban food systems by answering the following statement: “Urban food systems contribute to…”. Value scale from 1 – strongly agree - to 5 –strongly disagree.

|  | **1** | **2** | **3** | **4** | **5** | **DK** | **Total** |
| --- | --- | --- | --- | --- | --- | --- | --- |
| **Provision of medicine and aromatic plants** | 275 | 66 | 20 | 10 | 3 | 1 | 375 |
| **Provision of food** | 262 | 74 | 30 | 12 | 2 | 0 | 380 |
| **Facilitation of pollination** | 167 | 111 | 40 | 15 | 10 | 35 | 378 |
| **Provision of other raw materials** | 172 | 90 | 49 | 23 | 31 | 12 | 377 |
| **Facilitation of biological control** | 154 | 82 | 45 | 18 | 21 | 59 | 379 |
| **Conservation of genetic variability** | 97 | 86 | 43 | 22 | 12 | 116 | 376 |
| **Habitat for animals** | 168 | 93 | 42 | 17 | 11 | 47 | 378 |
| **Improvement of air quality** | 163 | 86 | 39 | 31 | 21 | 38 | 378 |
| **Increase of global biodiversity** | 92 | 68 | 49 | 21 | 55 | 94 | 379 |
| **Improvement of local climate** | 114 | 83 | 48 | 38 | 29 | 67 | 379 |
| **Regulation of the urban metabolism**  **(e.g., waste, water and nutrient flows)** | 81 | 81 | 39 | 13 | 39 | 126 | 379 |
| **Carbon sequestration and storage** | 126 | 64 | 28 | 21 | 28 | 112 | 379 |
| **Prevention of soil erosion and maintenance of soil fertility** | 123 | 64 | 37 | 24 | 33 | 99 | 380 |
| **Reduction of the effects of extreme events (e.g., flood, hurricane)** | 114 | 61 | 34 | 17 | 45 | 107 | 378 |

F. Society and surrounding

1. Please, value the different ecosystem services associated to urban food systems by answering the following statement: “Urban food systems contribute to…”.

Value scale from 1 – strongly agree - to 5 –strongly disagree.

| **Socio-cultural services** | **1** | **2** | **3** | **4** | **5** | **DK** | **Total** |
| --- | --- | --- | --- | --- | --- | --- | --- |
| **Contribution to political realization** | 163 | 25 | 11 | 1 | 0 | 0 | 200 |
| **Maintenance of traditional knowledge and cultural heritage** | 212 | 61 | 20 | 17 | 28 | 39 | 377 |
| **Improvement of urban tourist attraction** | 184 | 87 | 57 | 21 | 9 | 18 | 376 |
| **Placement attachment** | 240 | 78 | 31 | 10 | 7 | 11 | 377 |
| **Community building** | 231 | 93 | 25 | 10 | 5 | 15 | 379 |
| **Urban aesthetics and cultural/art inspiration** | 256 | 85 | 15 | 7 | 3 | 13 | 379 |
| **Social cohesion** | 244 | 93 | 23 | 4 | 2 | 13 | 379 |
| **Improvement of Physical health** | 211 | 91 | 33 | 11 | 4 | 28 | 378 |
| **Improvement of physical health** | 211 | 107 | 29 | 9 | 4 | 19 | 379 |
| **Contact with nature and artistic expression** | 226 | 95 | 30 | 5 | 6 | 16 | 378 |
| **Education and training** | 232 | 93 | 23 | 8 | 6 | 17 | 379 |
| **Recreation and enteratinment** | 192 | 60 | 29 | 21 | 32 | 44 | 378 |

1. Do you think that UA can improve the image of your city?

| **Option** | **Respondents (n)** |
| --- | --- |
| **Large improvement** | 182 |
| **Improvement** | 177 |
| **Without effect** | 17 |
| **Deterioration** | 0 |
| **Large deterioration** | 0 |
| **DK** | 1 |
| **NA** | 3 |
| **Total (n)** | 380 |

G. Participation

1. Could you imagine to participate in UA? How? (multiple entries possible)

|  | **Yes** | **No** | **NA** | **Total** |
| --- | --- | --- | --- | --- |
| **I won't participate** | 9 | 233 | 138 | 380 |
| **Other** | 12 | 232 | 136 | 380 |
| **Funding** | 42 | 208 | 130 | 380 |
| **Food distribution** | 52 | 204 | 124 | 380 |
| **Organization of events** | 106 | 164 | 110 | 380 |
| **Food production** | 111 | 169 | 100 | 380 |
| **Education & trainning** | 173 | 143 | 64 | 380 |
| **Food acquisition** | 244 | 82 | 54 | 380 |
| **Relax space** | 271 | 86 | 23 | 380 |

1. In which area would you participate or contribute in an UA project?

| **Option** | **Respondents (n)** |
| --- | --- |
| **City-wide** | 157 |
| **Neighbourhood** | 200 |
| **Total** | 357 |
